# The embryonic role of juvenile hormone in the firebrat, *Thermobia domestica,* reveals its function before its involvement in metamorphosis

**DOI:** 10.1101/2023.10.06.561279

**Published:** 2023-10-10

**Authors:** James W. Truman, Lynn M. Riddiford, Barbora Konopová, Marcela Nouzova, Fernando Noriega, Michelle Herko

**Affiliations:** 1 Friday Harbor Laboratories, University of Washington, Friday Harbor, WA, USA; 2 Department of Biology, University of Washington, Seattle, WA USA; 3 University of South Bohemia, Ceske Budejovice, Czech Republic; 4 Biology Centre, Czech Academy of Sciences, Institute of Parasitology,Ceske, Czech Republic.; 5 Department of Biological Sciences and BSI, Florida International University, FL ,USA.; 6 Department of Parasitology, University of South Bohemia, Czech Republic.

**Keywords:** juvenile hormone, ecdysone, myoglianin, metamorphosis, differentiation, precocene

## Abstract

Juvenile hormone (JH) is a key regulator of insect metamorphosis. To understand its role before metamorphosis originated, we studied JH action in the ametabolous firebrat, *Thermobia domestica.* JH levels peak late in embryogenesis and are low through early juvenile stages. Chemical suppression of embryonic JH synthesis by 7-ethoxyprecocene blocks embryonic differentiation, but the latter is restored with exogenous JH. Premature exposure of younger embryos to JH suppresses growth and morphogenesis and the expression of morphogens, such as myoglianin. These embryos switch to premature differentiation as shown by muscle formation and synthesis of cuticle like that of later embryonic stages.

We hypothesize that this ancestral role of JH in supporting tissue differentiation was later exploited for the evolution of metamorphosis. In embryos, the temporal separation of morphogen signaling and JH secretion results in morphogenesis preceding differentiation. With the evolution of metamorphosis, embryonic morphogen systems were redeployed during juvenile growth for morphogenesis of imaginal primordia. JH was also redeployed, but it now occurred with morphogen signaling. This co-occurrence resulted in JH maintaining a juvenile quality to the bud, which the morphogens positive allometric growth. The disappearance of JH late in growth then allowed the unantagonized morphogens to drive the primordia into metamorphosis.

## INTRODUCTION

The evolution of insects from an obscure group of soil arthropods to the dominant meso-sized group in terrestrial and freshwater environments was accompanied by profound changes in their life histories. The first life history change involved modifications to postembryonic growth that supported the evolution of wings and power flight (Kukalová-Peck, 1978; Belles, 2019). These modifications brought about a hemimetabolous lifestyle that included the molt to the adult becoming a terminal molt and metamorphic changes during this adult molt when the articulated, functional wings were finally manifest. The subsequent step to complete metamorphosis involved a redirection of embryogenesis so that the insect that hatched from the egg was not a miniature version of the adult, but had a modified, larval body that was adapted to feeding and growth. The transition from larva to adult then occurred through a transitional stage, the pupa, thereby providing the three-part life history diagnostic of the “complete metamorphosis” exhibited by holometabolous insects (reviews: Jindra, 2019; Truman & Riddiford, 2002, 2019).

Classically, juvenile hemimetabolous stages were called nymphs and juvenile holometabolous instars were called larvae. Although this terminology is somewhat controversial, we will use it throughout this paper. Both nymphs and larvae depend on a sesquiterpene hormone, juvenile hormone (JH), to maintain their respective forms. JH has two essential functions: (1) it is required at the start of an ecdysteroid-induced molt to maintain the insect in its current stage (Riddiford, 1976), and (2) its presence during the intermolt periods ensures that imaginal discs and primordia remain dormant and to not begin premature morphogenesis to the adult stage (Truman et al., 2006). Besides its effects in regulating metamorphosis, JH is also involved in shaping polymorphisms such as those seen in the castes of social insects (Nijhout and Wheeler, 1982).

Although JH has profound postembryonic functions in maintaining the juvenile form in the more derived insect orders, its function in insects of the two ametabolous orders, the Archaeognatha (jumping bristletails) and the Zygentoma (silverfish), is poorly understood. JH is clearly involved in egg production in the firebrat *Thermobia domestica* (Bitsch et al., 1985). Its only reported effect on postembryonic development, though, is the ability of JH application to suppress scale formation during the molt from the third to the fourth juvenile stage (Watson, 1967). In crustaceans, the immediate precursor to JH, methyl farnesoate (MF), regulates ovarian maturation (Laufer, et al., 1998). Since insects and other Hexapoda (i.e. Collembola, Protura and Diplura) evolved from crustaceans (Regier et al. 2005; von Reumont et al. 2012), it has been proposed that MF and JH may have originally been involved in regulating reproduction and were subsequently captured to control metamorphosis (Goodman and Granger, 2005)

While JH has only mild effects on postembryonic development in *Thermobia* (Watson, 1967), treatment of eggs with JH produces severe developmental abnormalities (Rohdendorf and Sehnal, 1973). These severe developmental effects suggest that the developmental role of JH in insects was initially confined to the embryonic domain and it only later became involved in postembryonic development as life histories became more complex. Out study focuses on the role of JH during embryogenesis of the *Thermobia domestica*. By detailed examination of the effects of JH agonists and an antagonist on *Thermobia* embryos, we find that JH has an essential role of supporting terminal differentiation and maturation of the embryo. This ancient ability of JH to promote and maintain cellular differentiation during embryogenesis, then appears to have been extended into postembryonic life where it acts to antagonize morphogenic and allow the maintenance of a juvenile state until the animal has undergone sufficient growth to permit metamorphosis.

## RESULTS

### The time-course of embryogenesis in Thermobia

**Figure 1.**
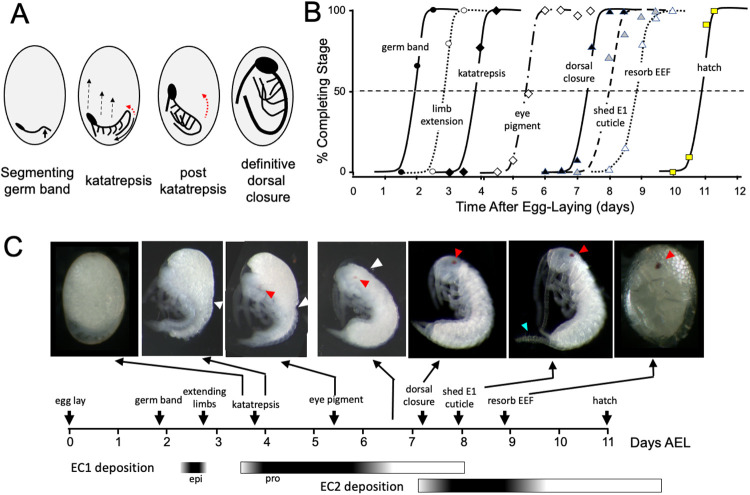
Timeline of *Thermobia domestica* embryogenesis at 37°C. **(A)** diagrammatic representations of important events in embryogenesis. In the segmenting germ band, the arrow indicates the invagination of the mid abdominal region into the yolk. By the onset of katatrepsis (Panfilio, 2008), dorsal closure along the abdomen (red arrow) has moved the abdomen ventrally (solid black arrow) and the contraction of the serosa pulls the sides of the embryo towards the anterior pole (dashed black arrows). After katatrepsis the expanding lateral edges of the embryo displace cells of the amnion, gradually zipping up the dorsal thoracic midline (red arrow) until definitive dorsal closure is accomplished. **(B)** Summary of the progression of embryonic development at various times after egg deposition. The interception of each stage with the 50% line (dashed) is the basis for the embryonic time-line in (C). EEF: extraembryonic fluid. **(C)** Timeline of embryonic development of *Thermobia domestica* based on (B). Photomicrographs show the appearance of embryos at the indicated times; first and last embryos are covered by the egg chorion. White triangle: progression of dorsal closure; red triangle: eye pigmentation; blue triangle: expanded cerci after shedding of the E1 cuticle. Times of cuticle deposition based on Konopová and Zrzavý, 2005.

The embryogenesis of *Thermobia domestica* is summarized in Fig. 1. At 37°C, the time from egg deposition to hatching is about 11.5 days. A developmental timetable was constructed using 12 hr egg collections and determining the distribution of developmental stages at half day intervals from the midpoint of each collection window (see Methods). Ages are given in days after egg laying (# d AEL). As with other insects from ametabolous orders, *Thermobia* embryos undergo short germ band development (Jura, 1972). A small embryonic rudiment forms at the posterior pole of the egg and consists of head and thoracic segments with a terminal growth zone. As abdominal segments are then progressively specified, the abdominal ventral midline invaginates into the yolk causing the abdomen to fold onto itself and opposing the ventral surfaces of the anterior and posterior abdominal segments (Fig 1A). By about 1.5 d AEL, the forming embryo is evident as a cleared slot at the posterior pole of the egg, and by a half day later abdomen formation is finished and buds for the segmental appendages are appearing. At this point the dorsal surface of the embryo is completely open to the yolk and the embryo has an overall S-shape (Fig 1A).

The embryo then begins “zipping up” its dorsal midline starting at the end of the abdomen and progressing to the juncture of the abdomen with the thorax. This dorsal closure of the abdomen pushes the abdominal tip ventrally and forward as the embryo with its yolk assumes a “comma” shape. Appendage buds are growing but have not yet reached half of their final extension by this time. Extraembryonic membrane growing up from the lateral sides of the embryo envelop the yolk mass and then, during katatrepsis, they contract to pull the head of the embryo towards the anterior pole and the embryo rotates to achieve a C-shape. The complete enclosure of the yolk by the extraembryonic membranes is termed “provisional dorsal closure” and for the next four days the lateral margins of the embryo gradually extend dorsally, replacing the cells of the amnion and achieving “definitive dorsal closure” when the embryo is finally completely enclosed in its own epidermis (about 7.5 d AEL). The period between katatrepsis and dorsal closure involves organogenesis, tissue growth, and cell determination. The embryo reaches its full size at definitive dorsal closure and then undergoes terminal differentiation and maturation during the last 4 days of embryogenesis.

Embryogenesis is accompanied by two bouts of cuticle production. The E1 cuticle is deposited after the formation of the segmented germ band. It contains little chitin (see below) and has a fibrous structure (Klag 1978, Konopová and Zrzavý, 2005) that presumably allows it to stretch to accommodate the subsequent growth of the embryo. The E2 cuticle starts to be deposited around dorsal closure as the embryo reaches its full size. The E2 procuticle has the lamellar structure (Konopová and Zrzavý, 2005) typical of insect cuticles (Neville, 1975) and it becomes a rigid covering for the hatchling. The latter is also called the first juvenile (J1) stage. It does not feed and sheds its E2 (=J1) cuticle a day after hatching to start its J2 stage. Feeding does not begin until it molts to the J3 stage.

### Endocrine events during embryogenesis of Thermobia domestica

**Figure 2.**
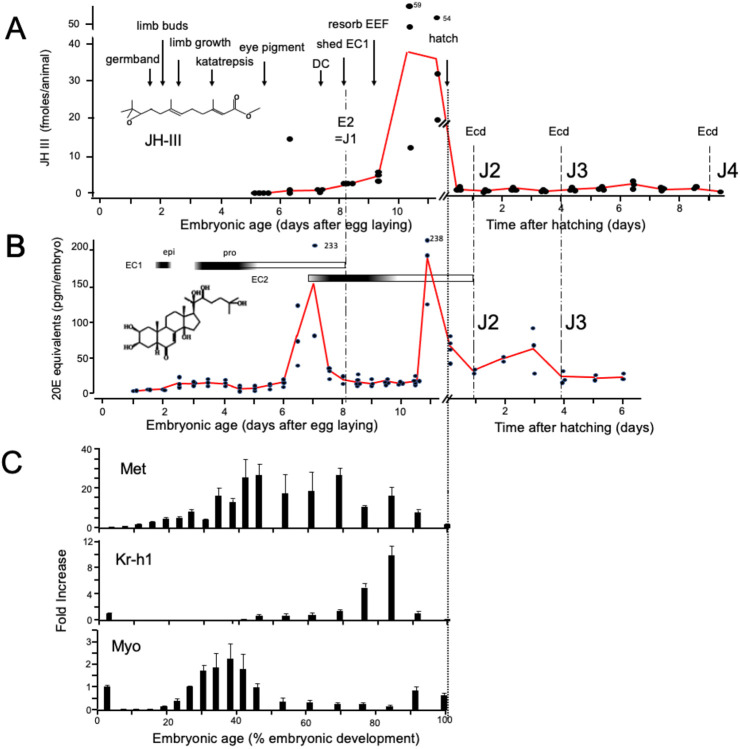
Titers of hormones and hormone related gene transcripts during embryogenesis of *Thermobia domestica*. **(A)** Titer of juvenile hormone III (JH-III) during the last 60% of embryogenesis and the first eight days of juvenile life. The timing of various milestones of embryonic development are noted for this panel and those below. ecd: ecdysis; J#: start of second, third and fourth juvenile instars. **(B)** The ecdysteroid titer in 20-hydroxyecdysone (20E) equivalents during embryogenesis and the first six days of juvenile life. Dark bars indicate the approximate time of deposition of the first embryonic (EC1) epicuticle and procuticle, and the second (EC2) embryonic cuticle (based on Konopová and Zrzavý, 2005). epi: epicuticle deposition, pro: procuticle deposition. **(C)** The relative levels of transcripts of the JH receptor, *Methoprene-tolerant* (*Met*), the JH response gene *Kruppel homolog 1* (*Kr-h1*), and the TGF-b family member *Myoglianin* (*Myo*) based on RT-PCR of stages through embryogenesis. The expression is related to the 12 hr timepoint which is given the value of 1. Each bar shows the mean (+/− S.D.) for three independent samples.

As in most insects, *Thermobia domestica* produces JH III (Baker et al., 1984). Using the Mass Spectrometric method of Ramirez et al. (2020), we measured JH III levels during *Thermobia* embryogenesis at daily intervals starting at 5 d AEL and extending through hatching and the first 10 days of postembryonic life (Fig. 2A). Detectable levels of JH III were found at 6 d AEL, and the titer slowly ramped up until an abrupt peak on days 10 and 11. After hatching JH III levels then fell to a low, but detectable, level through the start of the fourth juvenile instar.

Ecdysteroid titers through embryogenesis and the early juvenile instars were measured using the enzyme immunoassay method (Porcheron et al., 1989) that is optimized for detecting 20-hydroxyecdysone (20E). As seen in Fig 2B, there was a shallow, transient rise in ecdysteroid from 2.5 to 4 d AEL, associated with the time of deposition of the first embryonic (E1) cuticle (Konopová and Zrzavý, 2005). If *Thermobia* is like locusts (Lagueux *et al.*, 1977), then this first ecdysteroid peak would be expected to contain ecdysone, but no 20E. Ecdysone is poorly detected by the assay that we used and this low sensitivity for ecdysone likely explains the shallow nature of this first peak. A prominent peak of 20E then occurred around the time of dorsal closure, coordinated with the production of the E2 cuticle (= first juvenile cuticle), followed by another 20E peak just before hatching. This late embryonic peak is responsible for producing the J2 cuticle, that is seen when ecdysis occurs at 24 hours after hatching. The subsequent molt to the J3 stage is accompanied by an increase in 20E at about three days after hatching.

Fig. 2C summarizes the expression of some endocrine-related transcripts. Using RT-PCR (and the comparative Ct method; Schmittgen and Livak, 2008), we measured transcript levels at half-day intervals through the first half of embryogenesis and then at daily intervals thereafter. Transcripts for the JH receptor, *Methoprene-tolerant* (*Met*), appeared around the time of formation of the segmented germ band and extended through dorsal closure. *Met* transcript levels were reduced when the JH III titer spiked. *Krüppel homolog 1* (*Kr-h1*) is a highly conserved gene that responds to JH exposure (Konopova et al., 2011; Smykal et al., 2014; Belles, 2020; He and Zhang, 2022). Low levels of *Kr-h1* transcripts were present at 12 hr after egg deposition, but then were not detected until about 6 d AEL when JH-III first appeared. *Kr-h1* transcripts then spiked with the prominent peak of JH III at the end of embryogenesis. Members of the *Transforming growth factor-β* (*TGF-β*) family of morphogens (Upadhyay et al., 2017), notably myoglianin (myo), have become prominent as agents that promote the competence and execution of metamorphosis in holometabolous and hemimetabolous insects (He et al., 2020; Awasaki et al., 2011). We find that *myo* transcripts become abundant in *Thermobia* embryos after formation of the segmented germ band and as the limb buds are undergoing rapid growth and patterning but then decline to low levels by dorsal closure.

### Effects of suppression of the late embryonic peak of JH

**Figure 3.**
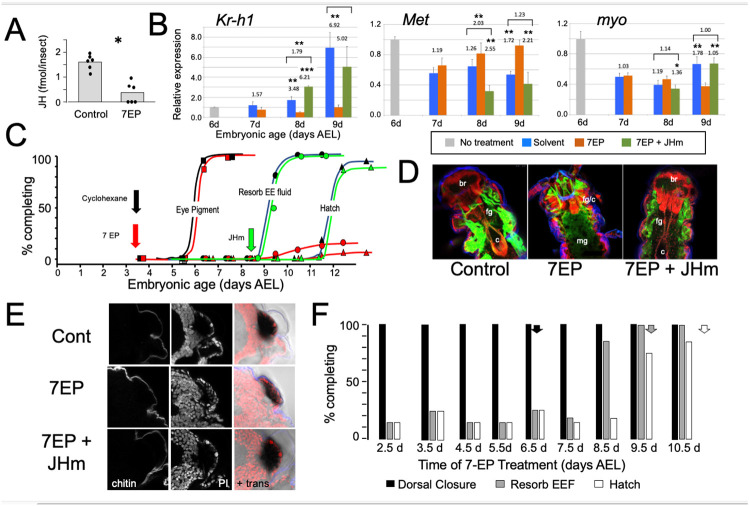
The effects of suppression of JH production on the embryonic development of *Thermobia*. **(A)** Compared with solvent alone (Control), treatment of stage 2 juveniles with 7-ethoxyprecocene (7EP) resulted in a >75% reduction in JH-III levels as measured 1 day later. **(B)** The effect treating embryos at 6 d AEL with the cyclohexane solvent (blue) or 1 μg 7EP (orange) on their subsequent expression of *Kr*-h1, *Met*, and *myo* over the next three days as revealed by RT-PCR. Sub-groups of 7EP treated embryos were treated with 1 ng JHm at 7 d and measured over the next two days (green). Significant differences determined by t-test: * = p < 0.05; ** = p < 0.01; *** = p < 0.001. **(C)** A group of 20 embryos were treated with cyclohexane (black) or 1μg 7EP (red) and their subsequent development monitored until hatching. Both groups started eye pigmentation at the same time, but resorption of the extraembryonic fluid and hatching was suppressed in the 7EP group, The latter two events were restored by treating embryos with the 1 ng JHm at 8.5 d AEL (green). **(D)** Confocal sections of the dorsal view of embryos treated as in “C” and examined at the time of hatching of the controls. 7EP treatment prevented extension of the foregut (fg) and crop (c) and the posterior displacement of the midgut (mg). Normal gut development was restored with JHm treatment at 8.5d. br: brain; muscle (green); propidium iodide staining (red). **(E)** Pseudo-transmitted light and confocal sections showing cuticle and nuclei of the eye region of 10 d embryos that had been treated with cyclohexane (control) or 7EP at 5 d AEL. A subset of the latter were given JHm on day 7.5. Control and JHm treated embryos show local apolysis of the eye cuticle and expansion of the depth of the eye due to growth of the rhabdoms; embryos treated with 7EP alone failed to show this growth. PI: propidium Iodide stain, + trans: pseudotransmitted light. **(F)** The relationship of the final phenotypes of embryos to the time of their treatment with 1 μg of 7EP. Arrows indicate the normal timing of the event. EEF: extraembryonic fluid.

Peak levels of JH occur towards the end of embryogenesis as tissues are undergoing maturation and terminal differentiation (Fig. 2A). To examine the role of this JH peak, we treated embryos with 7-ethoxyprecocene (7EP), a drug that suppresses JH production in many insects (Bowers and Martinez-Pardo, 1977; Aboulafia-Baginsky et al., 1984). As seen in Fig. 3A, treatment of young juvenile *Thermobia* with 7EP markedly suppressed their production of JH III. Also, application of 7EP to embryos at 6 d AEL, prior to the onset of JH secretion, prevented the JH-induced appearance of *Kr-h1* transcripts, but the latter could be induced by subsequent treatment with a JHm (Fig. 3B). Therefore, 7EP is effective in suppressing JH synthesis in *Thermobia*.

The effect of preventing JH production was first examined by treating embryos with 1 μg of 7EP at 3.5d AEL and then tracking aspects of subsequent development that could be viewed through the egg shell (Fig 3C). Solvent control and 7EP-treated embryos acquired eye pigment and underwent dorsal closure (not shown) at the same time, but the 7EP group failed to resorb their extraembryonic fluid and did not hatch. The latter two events, though, were restored to 7EP-treated embryos by application of JHm shortly after dorsal closure.

Dissection of the 7EP-treated embryos showed that they usually did not shed their E1 cuticle or expand their E2 cuticle. After dorsal closure, the foregut normally lengthens as the midgut is displaced posteriorly to the anterior abdomen, but these events did not occur after the 7EP treatment (Fig. 3D). On a tissue level, the eye primordium produces all the cell types for its twelve ommatidia by dorsal closure. These cell types attain their mature sizes by hatching and the overlying E2 (=J1) cuticle detaches in preparation for making the cuticular lenses of the J2 stage. This maturational growth of the eye and detachment of the cuticle was suppressed after the 7EP treatment (Fig. 3E). The normal maturation of both the eye and the midgut was restored in 7EP treated embryos by subsequent application of JHm (Fig. 3D, E).

Sensitivity to the suppression of JH synthesis by 7EP treatment did not end in an all-or-none fashion (Fig 3F). Development up through dorsal closure was unaffected, regardless of time of 7EP treatment, resorption of the extraembryonic fluid was suppressed by treatment up until about 8 d, and hatching was blocked until about 9 d AEL. This progressive loss of effectiveness of suppressing JH biosynthesis, suggests that JH does not provide a phasic signal, but rather acts tonically through the end of embryogenesis to bring about terminal differentiation and hatching.

**Figure 4.**
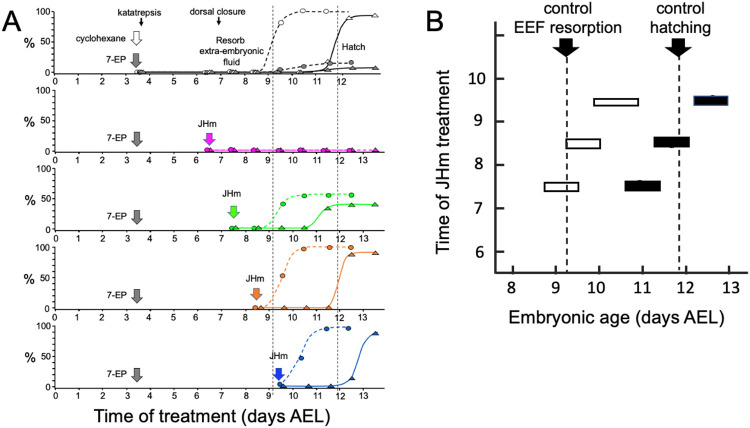
Time-course of the rescue of 7EP-imposed developmental arrest by treatment with JHm. **(A)** Groups of about 20 embryos were treated with cyclohexane (controls) or 1 μg 7EP at d3.5 AEL and then monitored daily for the time of reabsorption of the extraembryonic fluid and hatching. The vertical dashed lines indicate the 50% time for these two developmental events in the control group. Replicate groups were also given JHm (1 ng pyriproxyfen) at the indicated times and their development followed to hatching. **(B)** Summary of the timing of resorption of the extraembryonic fluid (white bars), as indicated by the appearance of air between the embryo and the eggshell, and of hatching (black bars) of 7EP-treated embryos that were then rescued by JHm treatment at the indicated times.

Figure 4A shows that the timing of JHm application was essential for rescuing the development block imposed by 7EP treatment. We recorded the time of the resorption of the extraembryonic fluid and of hatching for embryos treated with 7EP on 3.5 d AEL and then with a standard dose of JHm (1 ng of pyriproxyfen) at various days thereafter. Embryos that were treated with JHm at 6.5 d AEL (prior to definitive dorsal closure) were still in a phase of JH sensitivity (see below) and failed to complete dorsal closure. Day 7.5 AEL is about the midpoint for embryos undergoing dorsal closure (Fig 1). Half of the embryos treated at 7.5 d, did not complete dorsal closure and did not hatch. The remaining half had presumably undergone dorsal closure by the time of treatment and these subsequently resorbed their extraembryonic fluid and hatched. Essentially all the 7EP treated embryos given JHm on 8.5 d and 9.5 d AEL also showed fluid resorption and hatched. Importantly, the time of fluid resorption and hatching was time-locked to the time of the JHm treatment (Fig. 4A,B). Those treated at 7.5 d AEL hatched a day earlier than controls, those treated at 8.5 d hatched along with the controls, and those treated at 9.5 d showed a day delay in their hatching.

As expected, manipulation of JH levels had a marked effect on the levels of Kr-h1 transcripts. Suppression of JH production blocked induction of Kr-h1, while application of JHm to blocked embryos reinstated elevated Kr-h1 expression (Fig. 3C). Transcripts for *Met*, the JH receptor, show a mild down-regulation when JH III appears late in embryogenesis (Fig. 2C). This down-regulation was blunted when JH synthesis is suppressed by 7-EP treatment, but then enhanced by application of a JHm. Compared to their peak abundance at mid-embryogenesis, levels of *myo* transcripts are quite low going into the terminal stages of embryogenesis (Fig 2C). These levels are largely unaffected by late manipulations of JH, although there may be a slight inductive effect of JH evident at 9 d AEL (Fig. 3B).

### Effects of early treatment with juvenile hormone

Rohdendorf and Sehnal (1973) first showed that treating *Thermobia* embryos with natural JHs and a wide array of synthetic analogs produced a range of embryonic derangements. We initially tested the effectiveness of JH III and its precursor, methyl farnesoate (MF), as well as three commonly used JH mimics, methoprene, hydroprene, and pyriproxyfen (Goodman and Cusson, 2012). When applied at 3 d AEL, all produced a full range of embryonic abnormalities, depending on dosage (data not shown). Between the naturally occurring compounds, JH III was about 8-fold more potent than MF, with an ED_50_ for topical application of 60 ng/egg while MF had an ED_50_ of 480 ng/egg. The synthetic JH mimics were about 100 times more potent than JH III and were used for subsequent experimental treatments. We used pyriproxyfen (referred to as JHm) for all the experiments detailed below –the JH receptor was shown to have higher affinity for pyriproxifen than for JH III or methoprene (Charles et al. 2011, Jindra et al. 2015).

**Figure 5.**
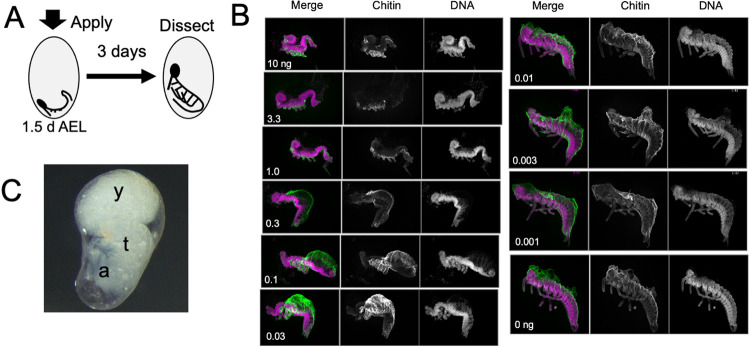
The response of *Thermobia* embryos to various doses of the JH mimic pyriproxyfen. **(A)** Cartoon showing the time of JHm treatment versus the time of dissection. **(B)** Lateral projections of confocal stacks showing the appearance of typical embryos three days after treatment with the indicated dosage of pyriproxyfen. Embryos were stained for DNA (magenta) and chitin (green). The embryos were dissected away from the yolk which caused disruption in the dorsal thoracic and head regions of some embryos. **(C)** Photomicrograph of an embryo arrested in mid-katatrepsis. During its envelopment, the contraction of the amnion segregated the yolk (y) from the embryo which hangs in the ventral half of the egg. t: thorax, a: abdomen.

Figure 5 examines the response of embryos to treatment of different doses of JHm at 1.5 d AEL, when the forming germ band can first be seen at the posterior pole of the egg. The embryos were then dissected at 4.5d AEL, a time after the completion of katatrepsis by control embryos. Embryos treated with the highest doses of JHm (1 to 10 ng/egg) always formed a complete segmented germband, with its various appendage primordia. However, the embryos retained an S-shaped posture and stayed at the posterior pole of the egg. Their growth was suppressed, and the embryos remained open dorsally from the head to the end of the abdomen. The cells of the amnion did not extend to enclose the yolk and these embryos often later sunk back into the yolk. Despite their early arrest, they secreted a cuticle over their ventral surface (chitin staining in Fig. 5B).

Embryos exposed to intermediate doses of JHm (0.3 to 0.03 ng pyriproxifen) were able to subsequently “zip up” the dorsal midline of their abdomen, which pushed the end of the abdomen ventrally and forward, causing the embryo to assume a “comma” shape just prior to the start of katatrepsis. Although the abdomen was able to undergo its dorsal closure, the subsequent morphogenesis of the head and thorax was suppressed. Cuticle that contained chitin covered the entire abdomen but only the ventral surface of the head and thorax. In many cases, the amnion did not spread dorsally to enclose the yolk and establish provisional dorsal closure. In cases in which provisional dorsal closure did occur, the arrested development of the embryo’s head and thorax prevented their lateral margins from being pulled laterally around the yolk, and the yolk ended up in a rounded dorsal lobe with the embryo hanging ventrally in a fluid-filled space (Fig. 5C).

Embryos treated with low doses of JHm (below 0.01 ng pyriproxifen) underwent normal katatrepsis and their amnion spread to cover the yolk to provide provisional dorsal closure. We did not track the development of this experimental group beyond 4.5 d AEL.

**Figure 6.**
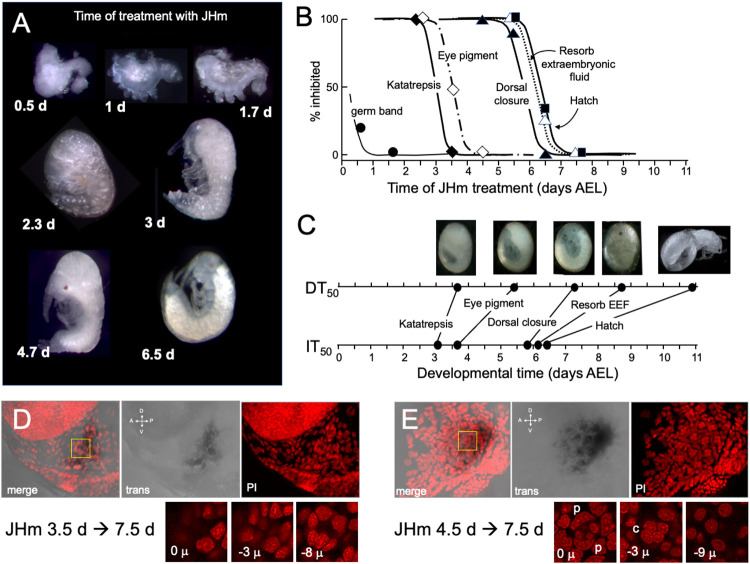
The response of *Thermobia* embryos to 1 ng JHm given at different times through embryonic development. **(A)** Examples of the terminal phenotypes of embryos resulting from JHm being applied at the indicated times [days after egg laying (AEL). Yolk was dissected away from the first three embryos. The remaining embryos had undergone provisional dorsal closure with the amnion enclosing the yolk mass. **(B)** Graph showing the times when the indicated event can no longer be inhibited by treatment with 1ng of pyriproxyfen (JHm). The 50% point defines the Inhibitory Time_50_ (IT_50_). **(C)** Comparison of the IT_50_ with the time when 50% of the embryos reach corresponding developmental milestones (Developmental Time_50_; DT_50_). **(D, E).** Lateral confocal and pseudotransmitted light images of the eye region of 7.5 d embryos that had been treated with JHm at (D) 3.5 d or (E) 4.5 d AEL. In (D) only the posterior quarter of the eye placode has been patterned as seen by the posterior crescent of screening pigment and formation of only 3-4 proto-ommatidial clusters. In (E) the entire eye placode has acquired its final form with its 12 ommatidial clusters. The insets below show various Z depths of the boxed cluster in the merged image, with “0 μ” being at the surface. Nuclei of identifiable ommatidial cell types can only be recognized in (E). c: quartet if crystalline cone cells, p: crescent-shaped nuclei of the paired cells that secrete the cuticular lens.

The phenotypes of JHm-treated embryos also varied with the time of application (Fig. 6A). Treatment of eggs within 12 hr of oviposition was problematical because the cyclohexane-treated controls often showed significant lethality. With older embryos, though, treatment with the solvent alone did not interfere with subsequent development. Figs. 6B, C summarize the ability of JHm treatment to suppress various of the “milestones” of embryonic development that we could observe through the egg chorion. Groups of embryos were treated with 1 ng pyriproxyfen at the indicated time, and we determined its effect on subsequent development. The developmental time at which JH treatment could no longer inhibit a given event in 50% of the embryos (the IT_50_) (Fig. 6B) could then be compared with the time when that event normally occurs in half of the embryos (the DT_50_) (Fig. 6C). Very early (12h AEL) treatment with JHm typically resulted in embryos that formed a segmented germ band, but they remained in the S-shaped form at the posterior pole of the egg. With later treatments with JHm, the embryos reached progressively later developmental milestones before they arrested. Although the DT_50_’s for these developmental milestones were fairly evenly distributed through the last 75% of embryogenesis, their IT_50_’s were in two clusters (Fig 6C). The late cluster included definitive dorsal closure, reabsorption of the extraembryonic fluid, and hatching. Embryos that fail to complete dorsal closure do not undergo the subsequent events. The attainment of definitive dorsal closure coincides with the ramping up of endogenous JH secretion by the embryo (Fig 2A) and, as shown above, the remaining events require JH for their completion. Supplementing the endogenous JH peak with additional JHm does not affect these late processes.

The early processes blocked by by premature exposure to JHm include katatrepsis itself. As described below, the presence of JHm during the E1 ecdysteroid peak causes the production of an altered E1 cuticle that may not be able to stretch and deform as the extraembryonic membranes contract, thereby aborting the process. The ID_50_ for eye pigment production occurred slightly later at 3.5d AEL (Fig. 6B,C). We think that the latter represents the time when the eye placode begins eye formation. Before this time, JHm treated embryos did form any recognizable eye elements. Treatment at 3.5d AEL resulted in half of the embryos subsequently showing the red screening pigment of a developing eye, while the other half did not. The former embryos, though, only produced a few ommatidia clusters at the posterior margin of the eye (Fig. 6D) and cell types within the cluster were not recognizable (although some are obviously making screening pigment). When JHm treatment was delayed to 4.5 d AEL, the full set of 12 ommatidial clusters were formed, and we could identify nuclei of specific cell types such as the two secondary pigment cells and the underlying quartet of corneal cone cells (Fig. 6E). Thus, we see that in *Thermobia*, like in other insects (Friedrich, 2006), the eye placode makes ommatidia progressively starting at the posterior border of the placode and progressing anteriorly. Depending on time of application, JHm can block either the initiation or the progression of this wave of ommatidia formation. Even after a pre-cluster has formed, JH can suppress its subsequent patterning and determination of cell types.

**Figure 7.**
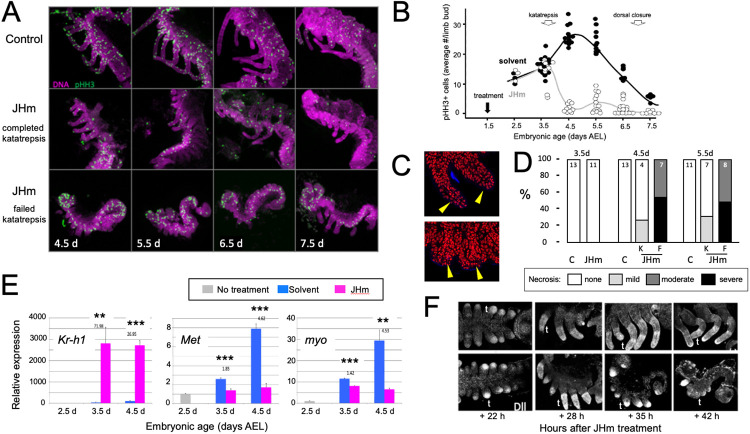
Effects of early treatment with JHm on the subsequent growth and patterning of the limbs. **(A).** Z-stack projections showing the lateral view of embryos that were treated with either cyclohexane (Control) or 1 ng of pyriproxyfen in cyclohexane (JHm) on d1.5 AEL and then dissected and stained at the indicated times thereafter. Propidium iodide (PI) (magenta) shows nuclei and anti-phosphohistone H3 (pHH3; green) shows dividing cells. The JHm series follows a subset of embryos that did not undergo katatrepsis (bottom) and a subset that did (middle). **(B).** Summary of the number of pHH3-positive cells in the developing limb buds of JHm-treated and control embryos through time. Each dot records the average number of pHH3-positive cells /limb from a single embryo. **(C).** Single optical sections through the limb buds of JHm-treated embryos that did not undergo katatrepsis and illustrating moderate (upper) and high (lower) levels of necrosis. Dead cells were evident as highly condensed PI-positive bodies (yellow triangles). **(D).** The time course of necrosis in the limbs of control and JHm-treated embryos. Necrosis was pronounced in embryos that failed katatrepsis (F) but rather mild in those that completed katatrepsis (K). **(E).** Levels of hormone-related transcripts in embryos treated at 2.5 d AEL with solvent alone (blue bars) or JHm (1 ng pyriproxyfen; pink) and then examined over the following two days. The expression is related to day 2.5, no treatment (grey) which was set as 1. *Kruppel homolog 1* (*Kr-h1*), Methoprene tolerant (*Met*), *myoglianin* (*myo*). Significant differences determined by t-test: * = p < 0.05; ** = p < 0.01; *** = p < 0.001. **(F).** Confocal images showing the effect of treating embryos at 2 d AEL with solvent (C) or JHm and subsequently immunostaining for Distal-less protein (Dll) at the indicated times thereafter. Embryos are shown from ventral view at 22h post-treatment, and then from lateral view thereafter. t: first thoracic leg.

The effects of premature exposure to JHm were further examined for embryos treated with pyriproxyfen at 1.5 d AEL and then *dissected* and immunostained at daily intervals thereafter. The embryos showed a range of responses to the JHm treatment, with different individuals arresting before, during or after katatrepsis. Figures 7A, B summarize the effects of JHm treatment on growth of the limb buds, assessed by number of mitotic cells revealed by expression of phosphohistone H3 (pHH3) (Hendzel et al., 1997). The growing limb buds were obvious in both groups at 2.5 d AEL; they were of similar size and showed similar levels of pHH3 expression (Fig. 7B). In control embryos, the number of pHH3+ cells peaked between 4.5 d and d5.5 d AEL, when their limbs had achieved their full length, and then declined. At 3.5 d AEL, most of the JHm-treated embryos showed numbers of pHH3+ cells like those seen in controls, although two embryos showed a marked reduction. By 4.5 d AEL, all the JHm treated embryos showed mitotic cell a frequency that was 10-15% of that seen in controls (Fig. 7B). Mitosis in their limbs remained low through subsequent development.

As summarized in Figs. 7C, D, the JHm treatment sometimes evoked necrosis in the growing limb buds. Necrotic bodies, consisting of highly compacted DNA, were rarely seen in the limbs of control embryos at 4.5d and 5.5d AEL. For the JHm-treated embryos, those that completed katatrepsis also showed few, if any, necrotic bodies, but those that arrested before or during katatrepsis exhibited moderate to heavy necrosis (Fig. 7C, D). In the most severe cases the limb bud completely degenerated. Limb loss in such embryos was often stochastic, *i.e.,* in a given embryo some limbs were completely lost while others were maintained in a reduced state. We saw no segmental pattern as to which limbs were maintained and which were lost.

The treatment at 2.5 d AEL with JHm also altered the subsequent expression of *Kr-h1, Met*, and *myo* (Fig. 7E). As expected from postembryonic effects in other insects, exposure to JHm induced strong *Kr-h1* expression, and mildly suppressed *Met* transcripts. Transcripts for *myo* normally increase as the limb buds are undergoing rapid growth from about 2.5 to 5d AEL (Fig. 2C), but JHm treatment prevented this increase (Fig. 7E), in parallel with its suppression of proliferation in the limb (Fig. 7B).

Early treatment with JHm also interrupted expression of the proximal-distal patterning of the forming limb. Limb bud formation requires the expression of *distal-less* (*dll*) at the tip of the growing bud (Cohen and Jürgens , 1989; Panganiban and Rubenstein, 2002). Up through the entry into katatrepsis, JHm-treated and control embryos showed similar expression of Dll in the distal half of the growing bud (Fig. 5F). As leg development progresses, recruitment of additional proximal-distal patterning genes resolves the *dll* expression into a “sock and band” pattern (*e.g.,* Angelini and Kaufman, 2005; Schaeper et al, 2013) as intermediate leg regions become specified. The JHm-treated embryos shown in Fig. 7F are ones that did not complete katatrepsis and they did not further refine their Dll expression. Dll immunostaining was eventually lost as their limbs degenerated. The JHm-treated embryos that completed katatrepsis, by contrast, maintain legs that were shortened and but possessed the appropriate segments. We assume that the latter embryos progressed far enough through the proximal-distal patterning program before the JHm-induced arrest to produce a stable leg that maintained its integrity. An earlier arrest of the patterning program, however, appears to be insufficient to maintain a stable leg.

### The effects of JHm on cellular differentiation

**Figure 8.**
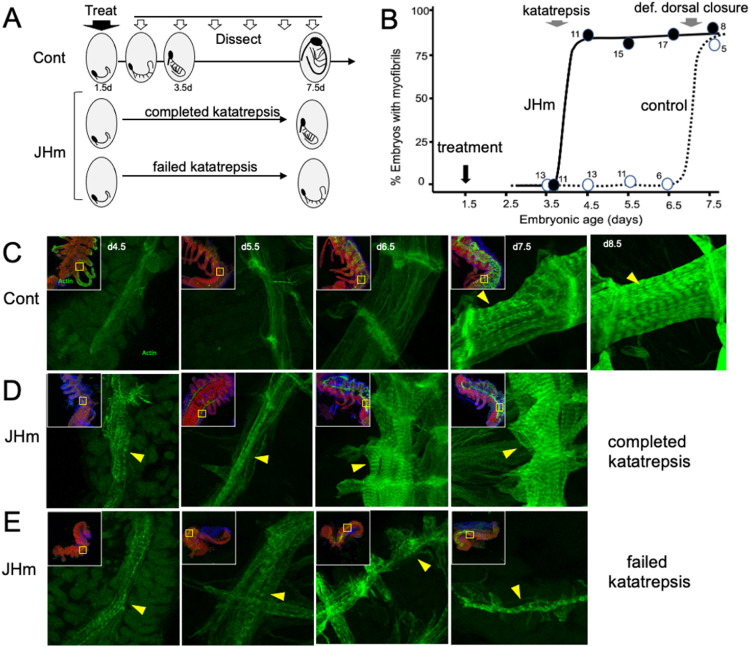
The effects of JHm in inducing early differentiation and terminal maturation in embryos of *Thermobia*. **(A).** Schematic showing the time of treatment with solvent or 1 ng pyriproxyfen (JHm) and the subsequent dissection and staining of the embryos. **(B)** Quantitation of the effects of JHm treatment on the appearance of striated myofibrils in the developing muscles of *Thermobia* embryos. Myofibrils normally appear around the time of definitive dorsal closure, but their appearance is advanced by three days in the JHm-treated embryos. **(C-E).** Confocal optical sections showing F-actin staining in developing longitudinal muscles in control (C) and JHm-treated (D,E) embryos from 4.5 to 8.5 d AEL. Yellow triangles indicate striations of the myofibrils. Insets show low power views of embryos with the magnified region boxed. green: F-actin shown by phalloidin binding, red: propidium iodide. JHm-treated examples are embryos that underwent katatrepsis (D) and ones that failed to complete katatrepsis (E).

While the early JHm treatment suppressed growth, patterning, and cell determination, it also induced cellular differentiation as illustrated by the forming musculature (Fig. 8). In control embryos (Fig. 8C), longitudinal muscle fibers are first evident in the thorax and abdomen on 4.5 d AEL as thin bands of tissue that show weak, uniform staining for actin (as revealed by binding of fluorescent-phalloidin). The developing muscles increase in width over the next two days and their actin staining concentrates at the intersegmental boundaries where the developing muscle fibers attach to tendon cells. The appearance of myofibrils with their repeating sarcomeres becomes evident by 7.5 d AEL, but such embryos, dissected from the egg shells, showed neither spontaneous nor induced movements. By a day later the embryos often showed weak, spontaneous movements when removed from the egg and their muscles have well developed striations (Fig 8C, 8.5d). By contrast, embryos treated with 1 ng of pyriproxyfen at 1.5d AEL showed accelerated muscle differentiation (Fig 8D, E). Treated embryos that did not undergo katatrepsis did not show subsequent growth but their longitudinal muscles produced myofibrils by 4.5 d AEL (Fig. 8E). Treated embryos that completed katatrepsis also showed the premature production of myofibrils (Fig. 8D) and they underwent substantial growth.

**Figure 9.**
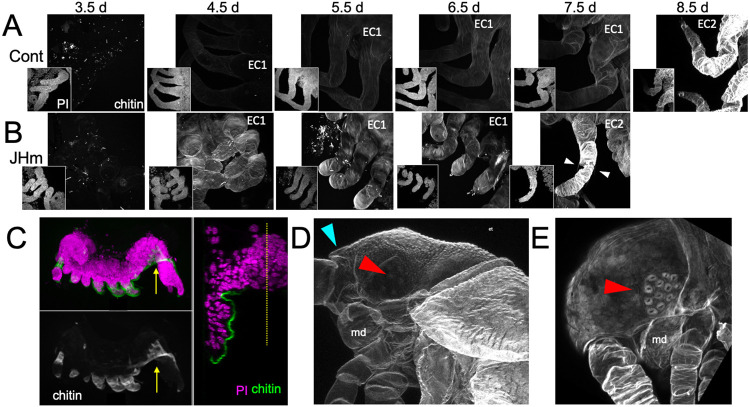
Effects of 1ng pyriproxyfen (JHm) on the production of E1 and E2 cuticles. **(A, B)** Projected confocal Z-stacks of embryos treated with solvent (Control) or JHm at 1.5d AEL and showing chitin staining of the developing legs at various times thereafter. The insets are PI staining of the same stack to show the form of the limbs on each day. In both groups no chitin was evident at 3.5d AEL, when only epicuticle is present (dots are due to surface debris). In control embryos, the procuticle of the first embryonic cuticle (EC1) (starting at 4.5 d AEL) stains weakly for chitin. The second embryonic cuticle (EC2) shows strong chitin staining (8.5d). JHm-treated embryos show enhanced chitin staining in their EC1 and they produce their EC2 a day early as demonstrated by the presence of cuticular hairs (white triangles). All cuticle images were made with the same laser power and gain. **(C)** Lateral view of a 5.5 d embryo that was treated with JHm on d1.5 and failed to undergo katatrepsis. The E1 cuticle covering its ventral surface contains higher than normal amounts of chitin staining. The arrow shows the plane of the transverse image the ventromedial region of the embryo is still invaginated into the yolk. PI: propidium iodide staining. **(D, E)** Projected confocal Z-stacks of untreated (D) and treated (E) embryos showing the effect of JHm treatment at 4.5 d AEL, prior to the production of the EC2 and examined around day 10. The cuticle of the control embryo (D) has a pebbly surface sculpturing, an egg tooth (blue triangle) but lacks cuticular eye lenses (red triangle). The JHm-treated embryo deposited a smooth cuticle that lacks an egg tooth but has abnormally formed cuticular lenses (red triangle). md: mandible.

JHm treatment had a marked effect on the cuticle produced by young embryos. In *Thermobia*, the epicuticle layer of the first embryonic cuticle (EC1) is secreted after germ band formation but prior to the start of katatrepsis (Fig 1; Konopová and Zrzavý, 2005). A fibrous procuticle is deposited after katatrepsis and covers the embryo through its period of rapid growth and dorsal closure. Chitin staining, using Calcofluor White (Harrington and Hageage, 2003), shows no chitin in the E1 epicuticle (as expected) (Fig. 9A, 3.5d), and only faint chitin staining of the procuticle (Fig 9A, 4.5 −7.5 d AEL), indicating that the latter is relatively poor in chitin fibers. Strong chitin staining comes with the deposition of the E2 procuticle on 8.5 d AEL. The ultrastructure of the latter shows a lamellar structure (Klag 1978; Konopová and Zrzavý, 2005), which arises from the helical deposition of chitin fibers in typical insect procuticles (Neville, 1965; Vincent, 1980). Like controls, embryos treated with JHm at 1.5 d had no chitin in their E1 epicuticle (Fig. 9B, 3.5d) but their E1 procuticle (Fig 9B, 4.5 d) showed enhanced chitin staining. The JHm-treated embryos also showed an advancement of at least a day in the deposition of their E2 cuticle with its bristles and hairs (Fig 9B, 7.5 d).

The JHm-treated embryos depicted in Fig. 9B were ones that completed katatrepsis. Figure 9C shows a treated embryo that failed to undergo katatrepsis but still secreted an E1 cuticle along its ventral surface. This cuticle also had enhanced staining for chitin. The modified E1 cuticle produced in the presence of JHm appears to be more rigid than the normal E1 cuticle. This rigidity may interfere with early embryos undergoing subsequent growth and changes in form.

JHm treatment before the formation of the E2 cuticle also changed the nature of this cuticle. As seen in Fig. 9D, the E2 (= J1) cuticle has a pebbly surface sculpturing, an egg tooth, and lacks cuticular lenses for the ommatidia. Those treated with JHm at 4.5 d AEL, by contrast make a cuticle that lacks the egg tooth and surface pebbling characteristic of the E1 stage but attempts to make cuticular lenses typical of the J2 instar (Fig. 9E). Indeed, the JH treatment redirects the molt to be more like that to the J2 stage, rather than to the E2 (= J1) stage. Precocious JHm exposure to embryos of locusts (Truman & Riddiford, 1999) and crickets (Erezyilmaz et al., 2006) likewise cause embryos to skip a stage.

Overall, then, embryonic development is sensitive to treatment with exogenous JH or JHm from after the formation of the segmented germband until definitive dorsal closure. JH treatment, though, does not simply arrest development. Rather, as shown by muscle and epidermis, JH switches the embryonic cells away from morphogenesis and into differentiation.

## Discussion

### *JH functions as a growth/differentiation switch in Thermobia domestica* embryos

**Figure 10:**
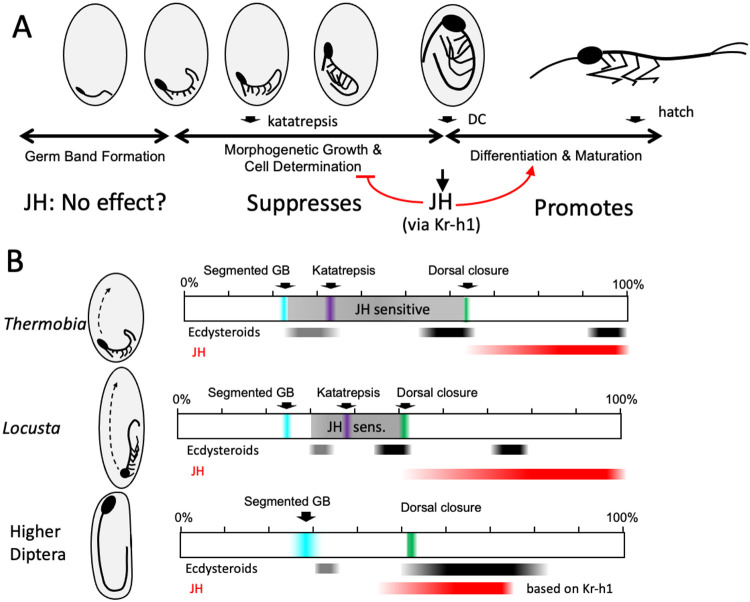
Comparison of the sensitivity of different embryos to JH. **(A)** The developmental responses to JH exposure vary through the embryogenesis of *Thermobia domestica*. Exogenous JH has little of no effect up through germ band formation. Coincident with the production of the first embryonic cuticle exogenous JH can suppress morphogenesis and evoke precocious differentiation. JH is normally released at definitive dorsal closure and supports terminal differentiation. **(B)** Comparison of the effects of exogenous JH on the development of different insects highlighting the importance of type of development (short germ band versus long germ band) and the time during development when ecdysteroids are first produced by the embryo. In the short germ band insects, *Thermobia* and *Locusta*, JH sensitivity is correlated with the ecdysteroid secretion that induces the first embryonic cuticle. Limb and body patterning is more advanced in *Locusta* versus *Thermobia* at that this initial molt, so its development is less repressed. Both embryos, though, undergo substantial body growth between the first molt and dorsal closure and this growth is suppressed in both. Long germ band embryos, by contrast, have finished their organ patterning by the time of the extended germ band and undergo body retraction, rather than growth as they progress to dorsal closure. They lack the intermediate growth period which is sensitive to JH in the short germ band embryos. *Locusta* ecdysone titers from Lagueux *et al*., 1977, and JH titers from Temin et al., 1986. Cuticle production and ecdysteroid titers in higher Diptera based on Calliphora (Bordes-Alléaume and Sami, 1987); JH inferred by embryonic expression of Kr-h1 (Beck et al. 2004).

Figure 10A summarizes the role of JH during embryogenesis of *Thermobia domestica*. Like in many other insects (Bergot et al., 1981b; Imboden et al., 1978; Temin et al., 1986), high levels of JH appear late in embryogenesis as the embryo undergoes terminal differentiation (Fig. 2A). The effects of blocking JH production, by treatment with 7EP, shows that terminal differentiation is, indeed, dependent on JH. This dependence is evident in the development of the compound eye (Fig 3E). The ommatidial units can form normally in the absence of JH, but they do not then progress into their mature configuration. The block of terminal maturation and hatching is restored to such embryos by treatment with JHm, in a manner that is linked to the time of JH treatment (Fig. 4B).

The role of JH in embryogenesis is further revealed by the effects of premature exposure of embryos to JH III or JHm. The diverse phenotypes observed after precocious exposure of embryos to JH (Figs. 5A, 6A) are consistent with the hormone inducing a premature shift from morphogenesis to differentiation. This is best seen in developing muscle (Fig. 8C-E); the myocytes of JH-treated embryos form banded myofibrils by 4.5 d AEL, rather than their normal time of 7.5 d AEL. Similarly in the epidermis, early JHM treatment induced an E1 cuticle that was chitin-rich and inextensible (typical of that deposited during the differentiation phase of embryogenesis), rather than the normal extensible, fibrous E1 cuticle (Fig. 9A,B). This premature differentiation was accompanied by the suppression of embryonic growth (Fig. 7A,B) and tissue patterning (Fig. 7F).

Premature exposure to JH also arrested katatrepsis and the progression of dorsal closure, processes that involve the amnion and serosa. It is not clear, though, whether these arrests result from a direct action of JH on these extraembryonic membranes, or if JH-induced changes in the embryo (such as secretion of a less extensible E1 cuticle) interferes with its response to contraction of the membranes.

Both experimental (Fig. 5E) and natural exposures to JH (Fig. 2C) are associated with stimulation of *Kr-h1* transcription. At present, we have only correlative evidence that JH works through Kr-h1 to suppress morphogenetic growth and promote differentiation in *Thermobia*, but the widespread involvement of Kr-h1 in diverse JH-mediated processes in insects (Jindra et al., 2015) make this highly likely. A target of JH, and likely Kr-h1, in *Thermobia* is myoglianin (Fig 7E). Levels of this morphogen peak during early morphogenetic growth (Fig. 2C) but are suppressed if embryos are treated with JHm (Fig. 7E). The correlation of myoglianin levels with limb growth in both normal and JHm-treated embryos (Figs. 7B versus E) is consistent with an involvement in the morphogenetic growth of the embryo. In *Drosophila* myoglianin acts systemically during the last larval stage to regulate imaginal disc growth so that they achieve a size appropriate for the insect (Upadhyay et al., 2020). Myo could be playing a similar role in *Thermobia* embryos to match limb growth to embryonic body size. The suppression of *myo* expression by JH may account for at least part of the growth suppression caused by early JHm treatment. Targets for JH and Kr-h1 undoubtedly go beyond myoglianin. Proximal-distal patterning in a limb (Fig. 7F) and the progressive patterning of ommatidial units in the eye (Fig. 6D,E) involves local, as well as systemic, morphogen signaling (Cagan, 2009). How JH may affect local morphogen signaling systems in *Thermobia* is unknown.

A circulating factor, like JH, that can shift the embryo from morphogenetic growth to differentiation might be especially important for a short germ band embryo that has to deal with a large extraembryonic yolk mass. In many insect species, egg size is not a constant and may vary with the size of the mother or with environmental conditions (Fox and Czesak, 2000; Yanagi and Midori, 2012). These variations may be especially challenging for short germ band embryos, such as those of *Thermobia*, in which the segmented germ band occupies only a small portion of the egg. The size of the embryo is not determined until the lateral regions of the embryo finally enclose the yolk mass at definitive dorsal closure. Since JH appears at this time, it likely provides a systemic signal that maximal growth has been achieved and embryonic processes should be shifted to terminal differentiation.

### Ecdysone and the onset of JH sensitivity in Thermobia embryos

An important question is when does the embryo first become sensitive to JH? Even with the earliest treatments with JHm, *Thermobia* embryos still form a segmented germ band with its appendage buds, although we cannot exclude the possibility that JH may have some subtle effects on this process. JH, though, definitely interacts with the first ecdysteroid peak to alter the first embryonic cuticle. In *Locusta migratoria*, this first embryonic ecdysteroid peak consists of ecdysone, but no 20E (Lagueux *et al*., 1977). This lack of 20E is not surprising because the principle tissues that normally convert ecdysone to 20E, such as the midgut, are just beginning to form. The composition of the first ecdysteroid peak in *Thermobia* is probably like that of *Locusta* and the fact that we detected only a slight rise in ecdysteroids at this time (Fig 2B) is likely due to the assay that we used being designed to detect 20E rather than ecdysone (Porcheron et al., 1989). Ecdysone is generally considered to be a “pro-hormone” but it does stimulate epidermal apolysis and cell division, while procuticle deposition occurs during the 20E portion of the ecdysteroid peak (Charles, 2009). The unusual characteristics of the E1 cuticle may be due to its induction by ecdysone alone. If so, then the early presence of JH may alter the composition of this first ecdysteroid peak or alter how the embryo responds to ecdysone. Unpublished studies of the effects of JHm on the early embryonic ecdysteroid peak in the locust, *Locusta migratoria*, show that JH does not change the composition of ecdysteroids (D.R.Williams, H.H. Rees, J.W. Truman and L.M. Riddiford, unpublished). Consequently, we think it likely that JH alters how the embryonic tissues respond to exposure to ecdysone alone.

A mechanism by which JH might alter the response of tissues to ecdysteroids is seen in the mosquito, *Aedes aegypti* (Liu *et al*., 2019). After emergence of the female, JH promotes the growth and maturation of the fat body, thereby preparing this tissue to respond to the ecdysteroids that are released after a blood meal. Ecdysteroids then act on the JH-primed fat body to induce synthesis and release of yolk proteins (Raikhel et al., 2005; Roy et al., 2018). An important molecular component for the actions of both JH and ecdysone is Taiman, a member of the P160 class of steroid hormone coactivators (Bai *et al.*, 2001). Taiman is involved in JH action as the heterodimeric partner with JH-Met, thereby forming an active receptor complex that binds to JH response elements of target genes such as *Kr*-h1 (Charles et al., 2011). In the ecdysone response pathway, Taiman is a major co-activator to mediate ecdysteroid activation (Montell, 2001). In *Aedes aegypti*, Taiman transcripts are alternatively spliced in a JH-dependent manner. While all isoforms are effective in mediating JH action, the two isoforms made in the absence of JH are ineffective in supporting ecdysone activation of fat body targets. JH, though, alters the splicing of *taiman* transcripts, thereby making two alternate isoforms that support strong steroid-induced activation (Liu *et al.*, 2019). We speculate that a similar relationship might hold for cuticle production in *Thermobia* embryos. Based on its actions during postembryonic molts, ecdysone likely is a weak activator of cuticle production in the embryo, perhaps leading to the unusual structure of the E1 procuticle. Exposure to JH, though, might alter *taiman* splicing, resulting in isoforms that allow ecdysone to mount a more robust molting response and a more mature type of cuticle. Regardless of the mechanism, though, JH clearly alters how the embryo responds to its first exposure to ecdysone.

### Variation in the response of insect embryos to early exposure to JH

The involvement of JH in terminal embryonic differentiation has been reported for other insects. Early experiments involving the ligation of embryos of the stick insect, *Clitumnus extradentatus*, to remove their corpora allata, resulted in the blockage of their terminal differentiation (Cavallin and Fournier, 1981). Subsequently, the use of precocene to suppress JH synthesis in embryos of the locust, *Locusta americana* (Aboulafia-Baginshy *et al.,* 1984), the cockroach *Nauphoeta cinerea* (Bruning et al., 1985) and the milkweed bug, *Oncopeltus fasciatus* (Dorn, 1982) also blocked late embryogenesis. In the latter two cases, the block was reversed by treatment with JH or JH mimics. Also, the use of maternal RNAi to knock-down components of the JH synthesis and response pathways interfered with late embryonic development in *Blattella germanica* (Fernandez-Nicolas and Belles, 2017) and the beetle *Tribolium castaneum* (Naruse et al., 2020). Genetic interruption of the JH pathway in *Bombyx mori* and *Drosophila melanogaster*, had little effect on embryogenesis although hatching was suppressed in the former (Daimon et al., 2015) and the latter showed interference in the late migration of the primordial germ cells to the somatic gonad (Barton et al., 2021).

Embryos of hemimetabolous insects respond to premature JH exposure similarly to those of *Thermobia*. In the locusts, *Schistocerca gregaria* (Novak, 1969; Injeyan et al., 1979) and *Locusta migratoria* (Truman and Riddiford 1999, 2002), the cricket, *Acheta domesticus* (Rao and Krishnakumaran, 1974; Erezyilmaz et al., 2006), and the true bug *Pyrrhocoris apterus* (Enslee and Riddiford, 1977), early treatment with JH or JHm blocks katatrepsis and results in nymphs with stunted appendages. In *Acheta domesticus*, treated embryos showed a marked reduction in tritiated thymidine incorporation (Rao and Krishnakumaran, 1974), indicating a suppression of proliferation, as we see in *Thermobia* (Fig 7A,B). The suppression of morphogenesis by early exposure to JH is accompanied by the precocious appearance of late developmental markers, e.g., sclerotized mandibles in *Acheta domesticus* (Erezyilmaz et al., 2006) and *Locusta migratoria* (Truman and Riddiford, unpublished) and advanced body pigmentation in *Pyrrhocoris apterus* (Enslee and Riddiford, 1977). Therefore, the ability of JH to shift embryogenesis from growth and morphogenesis to differentiation is shared by embryos of both ametabolous and hemimetabolous insects.

Two factors are involved in determining the severity of derangement of embryonic development in response to precocious exposure to JH or JH mimics. The first involves the relationship of the embryonic developmental timeline to the embryonic molts that are induced by episodic releases of ecdysteroids (Fig. 10B). A major ecdysteroid peak is consistently associated with definitive dorsal closure, providing the first cuticle to cover the embryo after its epidermis is completed and body growth is finished. The embryo also begins substantial JH secretion at this time, and additional, exogenous hormone has no effect. Earlier peak(s) of embryonic ecdysteroids occur after the formation of the segmented germ band but these are not tied to specific points in development. As summarized in Fig. 10B, the first ecdysteroid peak and production of the E1 cuticle occurs in *Thermobia* as its limbs are still extending tubes that show no demarcations between the various leg segments and have proximal-distal patterning genes in an early configuration (Fig. 7F). In locusts and crickets, by contrast, the comparable phase of ecdysone secretion and cuticle production (Lagueux *et al*., 1977, for *Locusta*) occurs relatively later in embryonic development when leg segments are clearly demarcated and limbs have been patterned (locusts: Truman and Riddiford, unpublished; crickets: Erezyilmaz et al., 2006). Hence, the most severe effect of precocious JHm treatment in the latter is an embryo with all of its leg segments, although stunted and somewhat malformed. In *Thermobia*, by contrast, the embryos arrest with incompletely patterned appendages that often then degenerate.

The second factor determining the severity of the arrest imposed by early JH exposure relates to the manner of germ band formation. Embryos of *Thermobia*, locusts, and crickets show short germ band development and have a period of substantial growth and morphogenesis until the time of definitive dorsal closure (Anderson, 1972a). Early JHm treatment suppresses this growth resulting in a tiny, arrested embryo at the posterior pole of the egg. Insects with intermediate germ band development (e.g., *Oncopeltus fasciatus* and *Pyrrhocoris apterus*) devote more of their blastoderm to making their embryonic rudiment. The differential between the body length of the germ band versus the completed embryo is less than seen in short germ band embryos as is the ability of precocious JH treatment to stunt embryonic growth (Enslee and Riddiford, 1977).

The extreme condition is long germ band development (Anderson, 1972b) seen only in some holometabolous groups such as Diptera and Hymenoptera. *Drosophila* provides an example of the latter in which the vast majority of the cellular blastoderm is devoted to making the embryo and it is simultaneously partitioned into the head and body segments. As the segmented germ band progresses into organogenesis, it becomes longer than the egg and folds back on itself. It also is establishing the primordia that will become the structures for the adult (Cohen, 1993). The germ band subsequently retracts to egg length before undergoing dorsal closure. Long germ band embryos lack a distinct phase of body growth between completion of the segmented germ band and terminal differentiation after dorsal closure. As such, they have lost the window of embryonic growth that is prominent in short germ band insects and is a major target of early JH exposure (Fig 10B).

Together, then, the changing strategies of germ band development interacts with the timing of secretion of ecdysteroids after formation of the germ band to determine how early embryos respond to precocious exposure to JH.

### JH and the progressive nature of embryonic molts

JH is classically known for its *status quo* action of maintaining the immature stages of hemimetabolous and holometabolous insects in their immature condition, and a progressive molt to the next life stage only occurs if JH is absent (Williams, 1961). The nature of the molt is determined at the beginning of the ecdysteroid peak and after this commitment phase (Riddiford, 1976), JH is no longer needed. In embryos of *Thermobia*, the ecdysteroid peaks that initiate the production of the E1 and E2 cuticles begin when JH titers are nil or extremely low (Fig 2B). In both cases, the early presence of JH redirects the molt in a progressive fashion to make a cuticle more typical of later life stages. For the E1 molt, for example, JH causes the epidermis to produce a chitin-rich, inextensible cuticle characteristic of later molts. The presence of JH at the start of the E2 (=J1) molt results in cuticle that lacks the pebbly surface sculpturing and egg-tooth of the E2 stage but has a smooth surface and partially formed cortical lenses characteristic of the J2 stage (Fig 9).

Premature progressive molts are also seen when locust (Sbrenna-Micciarelli, 1977 ; Truman and Riddiford, 1999) and cricket (Erezyilmaz et al., 2006) embryos are prematurely exposed to JH or JHm. The experimental application of JH prior to the onset of the E2 molt to the pronymph (also called the vermiform larva (Bernays, 1971) redirects embryonic development to form an embryo with proportions, cuticular pigmentation, cuticular sculpturing and bristles characteristic of a nymph, while pronymph modifications, such as the cuticular surface sculpturing (Bernays, 1971) and hatching teeth (Erezyilmaz et al., 2006), are missing. The tiny embryos that form after JHm treatment prior to the E1 molt of *Locusta* appear to secrete a more robust cuticle than normal, suggesting that JH can also modify the character of the E1 molt in orthopterans.

Although JH induces progressive molting in embryos of locusts and crickets, it switches to causing *status quo* molts in their nymphs. In many insects, the postembryonic effects of JH are largely mediated through Kr-h1. Since JH also induces Kr-h1 in embryos of many insects, including *Thermobia*, it is likely that the progressive actions of JH in the embryo are also mediated through the same transcription factor. How, then, does Kr-h1 mediate progressive molting during embryogenesis but then switch to causing *status quo* molts during the postembryonic period? One possibility is suggested from a comparison of action of JH in inhibiting metamorphosis of the nymphal locusts, with its reproductive actions in the adult of promoting fat body growth and maturation in the female (Wu et al., 2021). Both actions of JH are mediated through Kr-h1, but in the nymph, Kr-h1 recruits a co-repressor, C-terminal binding protein (CtBP), to suppress transcription of *E93*, thereby preventing transition to the adult. In the adult, by contrast, Kr-h1 recruits a coactivator, CREB-binding protein (CBP), to drive expression of Ribosomal protein L36 to support fat body growth. In a similar fashion, the cofactors available to Kr-h1 may change from the embryonic to the postembryonic period, shifting from coactivators that mediate the progressive molts of the embryo to co-repressors that maintain the *status quo* molts of the juvenile. A shift in availability of co-activators versus co-repressors may relate to the state of the tissue itself, *i.e.,* whether its cells are in an embryonic condition or are fully differentiated.

In locusts JH supports reproduction by causing the differentiation and maturation of tissues, like the fat body. It may, like in mosquitoes, also direct some aspects of the reproductive actions of ecdysteroids. Since the embryonic actions of JH in ametabolous and hemimetabolous insects involves similar themes of driving differentiation and enhancing ecdysteroid action, the reproductive and developmental functions of JH may have been very similar. The expansion of JH action into postembryonic molts in hemimetabolous insects then required a shift in Kr-h1 binding partners appropriate for maintaining the juvenile stage. The derived, postembryonic functions of JH is discussed in the last section.

### Modifications in JH function during the evolution of hemimetabolous and holometabolous life histories

The progressive evolution of new life history strategies from an ancestral pattern that was likely similar to *Thermobia* involved the extension of morphogenesis into postembryonic life. Morphogen systems, whose functions had been originally confined to embryogenesis, had to be redeployed during phases of postembryonic growth and a postembryonic function for JH apparently accompanied this redeployment.

**Figure 11.**
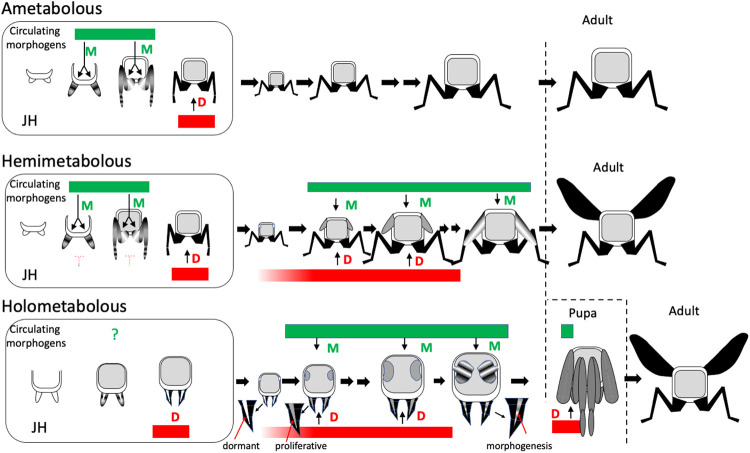
Proposed scheme for the evolving role of JH to support the invention of complex life histories In all three grades of development, various lines of evidence suggest that JH interacts with systemic morphogen(s) but a complete picture of the latter has yet to be finished. Overall, the morphogen systems are involved in morphogenesis (M) while JH promotes and supports differentiation (D). In the ametabolous condition the developmental functions of JH are confined to embryogenesis where morphogens and JH act at different times to support morphogenesis followed by terminal differentiation. For hemimetabolous development, morphogens reappear during the postembryonic period to support wing formation, but the extension of JH into the postembryonic period along with this morphogen signaling results in the formation of a nymphal wing bud rather than a wing. The disappearance of JH in the last nymphal stage then allows unantagonized morphogen signaling to form the wing structure. In the holometabolous pattern, embryonic changes bring about a modified larval body plan with much of morphogenesis deferred into postembryonic life. JH continues to be needed in postembryonic life to allow the controlled growth of imaginal primordia rather than their metamorphic morphogenesis. The latter can then occur when JH is removed. The differentiation of these imaginal primordia to their pupal state requires the return of JH during pupal differentiation, reminiscent of the embryonic requirement of JH for juvenile differentiation in embryos of more basal insect groups. See text for more detail.

Figure 11 summarizes the evolving role of JH as morphogenesis and morphogens are shifted into postembryonic life. The term morphogen encompasses many signaling systems with diverse functions in development. As seen for developing wing and leg imaginal discs in *Drosophila*, systems of secreted morphogens, such as *wingless/WNT* (Swarup and Verheyen, 2012), *hedgehog* (Ingham, 2022) and *decapentaplegic* (*TGF-β*) (Hamaratoglu et al. 2014) function locally within the wing to direct its morphogenesis, while others act systemically to coordinate wing growth with that of the animal as a whole. Of the latter, myoglianin, coming primarily from muscle, matches wing growth to body size (Upadhyay et al., 2020), while the relaxin-like growth factor, dILP8, coordinates growth between imaginal discs during formation of the pupa (Blanco-Obregon et al., 2022).

The morphogen systems of most importance to the role of JH likely involve circulating morphogens. Classic experiments by Piepho (1938) on the wax moth, *Galleria mellonella,* and by Wigglesworth (1934), on the bug, *Rhodnius prolixus*, showed that tissues from first instar caterpillars and bugs could be induced to start metamorphosis if exposed to the circulating environment of the last juvenile instar. The interpretation was that the JH-free environment of the last juvenile instar was sufficient to allow their metamorphosis, but experiments over a half century later from both holometabolous and hemimetabolous species (Daimon et al., 2015; Smykal et al., 2014) show that the simple removal of JH is not sufficient to cause first instars to initiate metamorphosis. These two sets of results can be reconciled by assuming that besides the lack of JH, the blood of the premetamorphic juvenile also has a factor(s) that promotes metamorphosis. Various hypothetical Metamorphosis Initiating Factors (Truman et al., 2006) and competence factors (Smykal et al., 2014; Chafino et al., 2019) have been proposed. A promising candidate is myoglianin. In *Manduca*, an increase of myoglianin release, likely from the muscle, signals that the larva has reached its critical size for metamorphosis, and the knockdown of myoglianin in *Tribolium casteneum* results in a permanent larva that continues to molt well after it has surpassed its critical weight (He *et al.,* 2019). In *Drosophila* myoglianin acts after the critical weight checkpoint in the last larval stage to match imaginal disc size to body size (Upadhyay et al., 2020) and to induce the ecdysone receptor isoform needed for neurons to start metamorphosis (Awasaki et al., 2011). While myoglianin is clearly a key player in preparing the juvenile stage for metamorphosis, it is not known if it is also the early competence factor, or if multiple morphogen systems were involved in evolving metamorphosis. Figure 11 refers to them simply as “circulating morphogens”.

The species-typical body plan is formed during embryogenesis in both ametabolous and hemimetabolous insects, and JH functions similarly in the embryos of both: it appears after dorsal closure to support the shift from morphogenetic growth to differentiation. Except for a possible involvement in the appearance of scales in the fourth instar juvenile (Watson, 1967), JH appears to have little or no role in the postembryonic development of *Thermobia*. Using levels of Kr-h1 as a proxy for the JH titer, Kr-h1 levels are low through juvenile growth and there is no dip in Kr-h1 levels during the transition to the adult stage (Fernandez-Nicolas et al., 2023). Nor is there evidence that JH is involved in sex-specific differentiation (production of genitalia) in *Thermobia*. A lack of JH involvement in the transition to the adult at the end of juvenile growth in *Thermobia* may not be surprising because such transitions are evident in all classes of Arthropods and, therefore, predates the emergence of JH.

The shift to a hemimetabolous life history involved postembryonic changes that resulted in the development of wings and of powered flight. The fossil record shows that the earliest winged insects were ametabolous with articulated winglets in the juvenile stages, but the wings showed positive allometric growth so that they were eventually became large enough to support flight (Kukalová-Peck, 1978). The postembryonic formation of the wing presumably involved the recruitment of some of the embryonic morphogen systems to function postembryonally in the thorax. To protect the forming wings from damage, later insects modified early wing growth so that it occurred as immobile wing pads, laid against the thorax. The transformation of the pads into articulated, functional wings was deferred until the molt to the adult stage. As suggested in Fig 11, the formation of the wing bud may have come about by the reappearance of JH during nymphal life. During embryogenesis, morphogen signaling and JH are separated in time so morphogenesis occurs before differentiation. In the hemimetabolous nymph, though, morphogen signaling and JH are finally found together. The actions of the morphogens to drive the formation of a wing would then be antagonized by the action of JH in maintaining cells in the differentiated, nymphal state. We think that the antagonistic interaction of the two systems resulted in a novel structure – the wing bud. The morphogen signaling maintain the bud cells as diploid and support their positive allometric growth, while the presence of JH maintain the nymphal state of the wing bud epidermis, so that it produces a nymphal cuticle and undergoes cell division only at the molts in response to ecdysone. This state requires the tonic presence of JH and either the experimental or natural loss of JH releases the break on the morphogen systems and the bud cells partially dedifferentiate and undertake the morphogenetic growth and patterning that results in wing production at the next molt. While the challenge of developing wings was likely the driving force that brought JH into the postembryonic realm, once JH was established in the nymphal stage, it was likely captured by other tissues to control their stage-specific differences between larva and adult.

The subsequent shift to the holometabolous lifestyle involved a fundamental change in embryogenesis to make the altered body plan of the larva (Fig. 11). As discussed above, the embryonic primordia of holometabolous insects could be divided into a portion used to make the larval version of their structure while the remainder is set aside to later become an imaginal primordium or imaginal disc (e.g., Cohen, 1993). These persisting embryonic primordia join the wing primordia in delaying their morphogenesis into postembryonic life. As proposed for the wing pads of hemimetabolous nymphs, the activation of imaginal primordia in holometabolous larvae likely also depends on morphogens. In newly hatched caterpillars of the butterfly *Pieris rapae*, each wing primordium appears as a thickened placode attached to the larva cuticle and persists as such into the second larval instar (Mercer, 1900). The placode has transformed into an invaginated imaginal disc by the beginning of the third instar, a similar time to when wing morphogenesis is first possible in the commercial silkworm (Daimon et al., 2015). The time of transformation of the placode into a disc produces likely indicates when key postembryonic morphogen systems become active, and it also is when JH becomes necessary for evoking premature morphogenesis. Studies in *Manduca sexta*, show that after this time the tonic presence of JH is required for the imaginal discs and primordia to grow in a nutrient dependent manner but maintains its larval differentiation. These primordia undergo dedifferentiation and start morphogenesis a few hours after the experimental removal of JH (Truman et al., 2006), presumably because the morphogens are no longer under JH suppression.

A unique feature of the JH titer in holometabolous insects is its transient reappearance during the formation of the pupal stage (Williams, 1961). Just before this time, ecdysone acts in the absence of JH to commit the larva to its a pupal fate and larval cells start to lose their larval specializations (Riddiford, 1976). During the subsequent prepupal ecdysteroid peak, the cells re-differentiate into their pupal form. For the cells of the imaginal primordia and imaginal discs, JH is often necessary for them to become pupal; and in its absence, they jump to the adult condition (Williams, 1961). This requirement of JH for pupal differentiation of imaginal disc cells is reminiscent of the need for JH by embryos of ametabolous and hemimetabolous insects to achieve their juvenile/nymphal condition. This postembryonic return of JH in the Holometabola, then, may accommodate the needs of embryonic tissues that have delayed morphogenesis and differentiation until metamorphosis. Also, it is interesting that this period when JH returns in *Drosophila* is also a time when another morphogen system involving dILP8 is signaling between imaginal discs to match their size to each other (Blanco-Obregon et al., 2022).

### Concluding speculations

Our results on *Thermobia* argue that the ancestral functions of JH in insects were not confined to reproduction. This hormone had an essential role during embryogenesis in supporting the shift from growth and morphogenesis to maturation and terminal differentiation. This function of JH in promoting/maintaining differentiation was then utilized to support the subsequent evolution of metamorphic strategies in the juvenile. The roots of JH signaling and action, though, extend back into the Crustacea where the immediate precursor of JH, methyl farnesoate (MF), is involved in reproductive control (Borst et al., 1987) but its effects on crustacean development are subtle and diverse (Laufer and Biggers, 2001) and difficult to compare with its developmental effects in *Thermobia*.

The functioning of farnesol derivatives in growth versus differentiation control extends deep into the eukaryotes. In the yeast *Candida albicans*, secreted farnesoic acid acts as a “quorum sensing signal” that controls the transition from a budding form to a hyphal form (Hornby et al., 2001; Oh et al., 2001). Amongst animals, the genes for the enzymes to synthesize farnesoic acid are widely found throughout the invertebrate phyla (So et al., (2022). In mammals, farnesol stimulates differentiation in epidermal keratinocytes (Hanley et al., 2000) while it also suppresses xenograft-based tumor growth in mice (Lee et al., 2015). Indeed, farnesol-based molecules may have an ancient involvement in switching cells between developmental states and this capacity was eventually exploited by the insects to provide the hormonal system that regulates their metamorphosis.

## METHODS

### Animals

The firebrats, *Thermobia domestica*, were reared at 37°C in small, covered polyethylene boxes. Layers of corrugated filter paper provided surfaces for the insects to move on and hide. The insects were fed dry Gerber’s baby rice cereal and a few pellets of dry cat food. Water was available in cotton-plugged vials. The incubator was kept above 70% relative humidity with a saturated KCl solution.

Cotton balls were placed between the filter paper sheets for oviposition. For timed egg collections, the cotton balls were replaced morning and evening to achieve 12 hour egg collections, and each was stored in a covered petri dish at 37°C until needed. The age of the embryos were referenced to the midpoint of the collection period.

### Juvenile Hormone Titers

Timed egg collections were extracted based on the method of Bergot et al. (1981a) as modified by Lacy Barton and Justina Sanny (personal communication) and by LMR. Briefly, 200 eggs were homogenized in 350 *μ*l acetonitrile containing 31.25 pg deuterated JH III (JHD3) in a silanized 2 ml Snap-It vial (Thermo-Fisher) on ice using a Benchmark D1000 homogenizer (Benchmark Scientific, New York), then capped and spun at 5000 g for 15 min at 4°C. The supernatant was transferred to a new silanized vial. The pellet was resuspended in 250 *μ*l acetonitrile containing 31.25 pg JHD3 III, spun at 5000g for 15 min, then the two supernatants combined. The combined supernatants were extracted with two volumes n-pentane and 4 volumes of 4% NaCl, then spun at 2000 g for 20 min at 4°C. The organic phases and aqueous phase were put into separate silanized vials, and the aqueous phase re-extracted with a half volume of n-pentane and spun 20 min at 4°C. The resultant organic phase was combined with the original organic phases which was then washed with an equal volume of 4% NaCl, and centrifuged at 2000 g for 20 min at 4°C. The organic phase was then transferred to a new silanized vial, the solvent gently blown off under a stream of nitrogen, and the residue then resuspended in 200 *μ*l acetonitrile. The residue and two 150 *μ*l rinses of the vial were loaded onto a Sep-Pak C18 column (3cc; Waters Corp., Milford, MA, USA). The purified extract was eluted from the column with 2 ml acetonitrile and frozen at −20°C until analysis by the liquid chromatography-tandem mass spectrometry method of Ramirez et al. (2020).

### Ecdysteroid titers

Ecdysteroid concentrations in timed egg collections were measured using the 20-Hydroxyecdysone (20E) Enzyme Immunoassay (EIA) kit (Cayman Chemical Company, Ann Arbor, MI) based on the method developed by Porcheron et al. (1989). Staged *Thermobia* embryos (200 per sample) were homogenized in 250 *μ*ls ice cold 75% aqueous methanol as outlined in Margam *et al*. (2006). Homogenized samples were centrifuged for 15 minutes at 13,000g and 4°C. Supernatants were transferred to 1.5 ml microcentrifuge tubes on ice. Pellets were resuspended in 250 *μ*ls 75% aqueous methanol, vortexed, placed on ice for 30 mins, then centrifuged as before, and the supernatants pooled. One quarter of the volume was dried down under nitrogen, stored desiccated at −20°C. It was resuspended in EIA buffer and diluted 1/100 just before assay. Following the kit instructions, the EIA was performed in a 96-well microtiter plate and is based on the competition between 20E (in standard or sample) and acetylcholinesterase-labeled 20E for the ecdysteroid antiserum that has been bound to the IGG-coated well plates. The plate was incubated with gyration overnight at 4°C, then washed five times with EIA buffer. The enzymatic substrate for acetylcholinesterase and chromogen, which form a yellow compound that absorbs between 405-414 nm, was then added to each well and incubated at room temperature with gyration for 60 min. The intensity of the color is inversely proportional to the amount of ecdysteroid present as determined by spectrophotometry on a Quant Microplate Spectrophotometer plate reader at 410 nm (BioTek Instruments, Vermont). The kit solutions were resuspended in UltraPure water (Cayman Chemical). The readings were compared to a standard curve derived from serial dilutions of the 20E standard and quality control provided by the kit.

### Hormones

Pyriproxyfen (Sumitomo Co.) and 7-ethoxyprecocene (Sigma Aldrich) were dissolved in cyclohexane (HPLC grade, Sigma Aldrich) and stored at −20°C. Using a 10 *μ*l Hamilton syringe, 0.2 *μ*l of hormone solution or of cyclohexane was applied with a repeating dispenser to staged eggs on double-stick tape.

### RNA analysis

Collections of timed embryos were placed in a 0.5 ml Eppendorf tube to which 150 *μ*l Trizol (Ambion) was added. The tube was vortexed, briefly spun, then frozen in liquid nitrogen. Total RNA was isolated using TRIzol (Invitrogen), treated with TURBO DNase (Invitrogen) and purified with RNA Clean & Concentrator (Zymo Research). cDNA was prepared from 2 *μ*g of the RNA using oligo-dT primer and SuperScript IV First-Strand Synthesis System (Invitrogen). PCR was performed using SsoAdvanced Universal SYBR Green Supermix (Bio-Rad); each 20 μl reaction contained 100 ng cDNA and forward and reverse primers, each primer at a final concentration of 500 nM. Reactions were run in the CFX Connect Real-Time PCR Detection System (Bio-Rad) and normalized expression was calculated using the associated software according to comparative Ct method (Schmittgen and Livak, 2008). The expression levels of each target gene were normalized against expression levels of the ribosomal protein 49 (rp49). Primers were designed using Primer3Plus (Untergasser et al. 2012) on the gene sequences that were derived from NCBI: Met and Kr-h1 were isolated by BK previously (Konopová et al. 2011), myo was found in the TSA database by blast search using Drosophila myo as a query and the amplicon sequence was also checked in the Thermobia genome (Brand et al. 2018) to check for nucleotide mismatches (sites of polymorphism). The accession numbers and primer sequences are in Figure 3 – Source Data 3. Melting curves were examined after each run and for each pair of primers several finished runs were visualized on a 2% agarose gel; only a single product was detected.

### Immunocytochemistry

Embryos were dissected and fixed in 3.9% formaldehyde (Thermo Fisher Scientific) in phosphate-buffered saline (PBS; Fisher Scientific) for 30 to 60 min, then rinsed three times in PBS with1% Triton X100 (PBS-TX; Fisher Scientific). They were then incubated with agitation in PBS-TX with various combinations of stains for 1-2 days at 4°C. Tissue stains were propidium iodide (Thermo Fisher Scientific, 1:1000 dilution of 1 mg/ml stock) or DAPI (Thermo Fisher Scientific, 1:1000 dilution of 1 mg/ml stock) for DNA, Alexa-488 conjugated phalloidin (Thermo Fisher Scientific, 1:100 dilution of 200 units/ml stock in methanol) for actin, and Calcofluor White (Sigma Chemical) for cuticle. Stained tissues were repeatedly rinsed, mounted on poly-L-lysine (Sigma-Aldrich) coated coverslips, dehydrated through a graded ethanol series, cleared through three changes of xylene, and mounted in DPX (Sigma-Aldrich).

A rabbit antibody against phosphohistone H3 (#06-570: anti phosphor-Histone H3 (ser 10); EDM Millipore, Darmstadt, Germany) was used at a 1:1000 dilution to identify mitotic cells. Tissue was preblocked in 2% normal donkey serum (Jackson Immunoresearch Laboratories, West Grove, PA, USA) for 15 to 30 min, then incubated with the primary antibody for two to three nights. Following 5 to 6 rinses in PBS-TX, tissues were incubated with a secondary antibody along with a variety of stains. Secondary antibodies were various Alexa Fluor 488-, 594-, or 647-conjugated donkey antisera raised against rabbit IgG fractions and used at 1:500 (Jackson Immunoresearch Laboratories, West Grove, PA, USA). Selection of the secondary depended on the combination of stains that were used. After staining, tissues were mounted, dehydrated, cleared and mounted as described above. The preparations were imaged on a Zeiss 800 confocal microscope and processed with ImageJ software (http://imagej.nih.gov/ij/).

## Supplementary Material

Supplement 1
