# Supplementary material for: The embryonic role of juvenile hormone in the firebrat, Thermobia domestica, reveals its function before its involvement in metamorphosis
Source: bioRxiv. 2023 Oct 10:2023.10.06.561279. Preprint. [Version 1] doi: 10.1101/2023.10.06.561279 (PMC10592639; doi:10.1101/2023.10.06.561279)
Supplement: Supplementary file 1 [file NIHPP2023.10.06.561279v1-supplement-1.pdf]

bioRxiv preprint doi: https://doi.org/10.1101/2023.10.06.561279; this version posted October 13, 2023. The copyright holder for this preprint (which was not certified by peer review) is the author/funder, who has granted bioRxiv a license to display the preprint in perpetuity. It is made available under aCC-BY 4.0 International license.

Source Data Tables:

Figure 1 __ source data 1. The percent of embryos attaining a particular developmental "milestone" as a function of the time after egg laying at 37°C. Age of each sample calculated from the midpoint of its 12 hr egg collection.

Embryonic age hrs (days)	N	germ band	extended limbs	post katatrepsis	eye pigmentation	dorsal closure	shed EC1	resorb EEF	hatch
50 h (2D)	23	65%	0						
62 h (2.5D)	23	100%	0						
74 h (3D)	24	100%	79%	0					
84h (3.5D)	25	100%	100%	0					
96 h (4D)	33	100%	100%	76%	0				
108 h (4.5D)	28	100%	100%	100%	0				
121 h (5 D)	24	100%	100%	100%	8%				
133 h (5.5 D)	23	100%	100%	100%	48%				
145 h (6 D)	23	100%	100%	100%	95%	0			
157 h (6.5 D)	38	100%	100%	100%	100%	0			
169 h (7 D)	30	100%	100%	100%	100%	10%	0		
180 h (7.5D)	48	100%	100%	100%	100%	77%	ND	0	
192 h (8 D)	37	100%	100%	100%	98%	98%	71%	3%	
204 h (8.5 D)	26	100%	100%	100%	100%	100%	85%	15%	
216 h (9 D)	41	100%	100%	100%	100%	100%	95%	78%	0
229 h (9.5 D)	28	100%	100%	100%	100%	100%	100%	96%	0
241 h (10 D)	50	100%	100%	100%	100%	100%	100%	100%	0
253 h (10.5 D)	50	100%	100%	100%	100%	100%	100%	100%	10
265 h (11 D)	50	100%	100%	100%	100%	100%	100%	100%	94%
277 h (11.5 D)	50	100%	100%	100%	100%	100%	100%	100%	100%

bioRxiv preprint doi: https://doi.org/10.1101/2023.10.06.561279; this version posted October 13, 2023. The copyright holder for this preprint (which was not certified by peer review) is the author/funder, who has granted bioRxiv a license to display the preprint in perpetuity. It is made available under aCC-BY 4.0 International license.

Figure 2 __ supporting data 1. Age of samples extracted for Juvenile Hormone III measurements. Age of samples from midpoint of 12 hr egg collections. E: embryonic age; J: age of juvenile; n.d.: below range of detectability.

Sample	Age at extraction	**individuals extracted**	**pg/ml**	**fmol**	**Fmole JH III/ individual**
001	E_5d 9h	196	n.d.	0	0
002	E_5d 9h	203	n.d.	0	0
003	E_5d 9h	205	n.d.	0	0
004	E_5d 9h	208	n.d.	0	0
005	E_6d 10h	202	97	54	0.5
006	E_6d 10h	212	5474	3082	14.5
007	E_6d 10h	191	265	149	0.8
008	E_7d 10h	205	265	149	0.7
009	E_7d 10h	134	119	67	0.5
010	E_7d 9hr	120	73	41	0.3
011	E_8d 9h	202	957	539	2.7
012	E_8d 9h	198	912	514	2.6
013	E_8d 9h	171	802	451	2.6
014	E_9d 9hr	198	1748	984	5
015	E_9d 9hr	192	1102	621	3.2
016	E_9d 9hr	199	1946	1096	5.5
017	E_10d 9h	204	4403	2480	12.2
018	E_10d 9h	201	15864	8933	44.4
019	E_10d 9h	201	21158	11914	59.3
020	E_11d 9h	191	10874	6123	32.1
021	E_11d 9h	209	7402	4168	19.9
022	E_11d 9h	167	16019	9021	54
030	J_d0 9hr	160	303	171	1.1
031	J_d0 9hr	150	125	70	0.5
032	J_d0 9hr	150	221	124	0.8
033	J_d1 9hr	150	58	33	0.2
034	J_d1 8hr	150	94	53	0.4
035	J_d1 8hr	150	51	29	0.2
036	J_d2 8hr	140	107	60	0.4
037	J_d2 8hr	131	104	59	0.5
038	J_d2 9hr	150	279	157	1

bioRxiv preprint doi: https://doi.org/10.1101/2023.10.06.561279; this version posted October 13, 2023. The copyright holder for this preprint (which was not certified by peer review) is the author/funder, who has granted bioRxiv a license to display the preprint in perpetuity. It is made available under aCC-BY 4.0 International license.

039	J_d3 9hr	150	46	26	0.2
040	J_d3 9hr	150	88	50	0.3
041	J_d3 9hr	140	53	30	0.2
042	J_d4 9h	75	85	48	0.6
043	J_d4 9h	75	103	58	0.8
044	J_d4 9h	75	121	68	0.9
045	J_d5 8h	75	109	61	0.8
046	J_d5 8h	75	169	95	1.3
047	J_d5 8h	75	89	50	0.7
048	J_d6 9hr	75	396	223	3
049	J_d6 9hr	75	383	215	2.9
050	J_d6 8 hr	75	76	43	0.6
051	J_d7 9 hr	75	139	78	1
052	J_d7 9hr	66	108	61	0.9
053	J_d7 9hr	66	73	41	0.6
054	J_d8 9hr	75	134	76	1
055	J_d8 9hr	75	156	88	1.2
056	J_d8 9hr	75	n.d.	0	0
057	J_d9 - J4	45	n.d.	0	0
058	J_d9 - J4	45	43	24	0.5
059	J_d9 - J4	40	n.d.	0	0

bioRxiv preprint doi: https://doi.org/10.1101/2023.10.06.561279; this version posted October 13, 2023. The copyright holder for this preprint (which was not certified by peer review) is the author/funder, who has granted bioRxiv a license to display the preprint in perpetuity. It is made available under aCC-BY 4.0 International license.

Figure 2 __ supporting data 2. Age of samples extracted for ecdysteroid measurements (given as 20 hydroxyecdysone equivalents). Age of samples from midpoint of 12 hr egg collections. Hatching occurred at 11.5 days after egg laying (AEL)

**age (days AEL)**	**Sample 1** pg/individual	**Sample 2** pg/individual	**Sample 3** pg/individual	**Sample 4** pg/individual	**Sample 5** pg/individual	**Average** pg/individual
1	4.2	7.1				5.7
1.5	6.8	7.7				7.2
2	8	7.1				7.5
2.5	23	8.8	11.9			14.6
3	11	11.7	14.8			12.5
3.5	9.5	13.4	19.2			14.1
4	10	11.5	17.1			12.8
4.5	5.9	8.2	9.6			7.9
5	8.5	6.8	10.6			8.6
5.5	7.4	12.2	14			11.2
6	15.8	9.6	18.9			14.7
6.5	39.5	74.7	128.1			80.7
7	82.6	233				157.8
7.5	34.3	35	20.8			30.1
8	14.84	23				18.9
8.5	13.2	11	12.4	19.8	29	17
9	12.2	20	11			14.4
9.5	12.2	20	14.2	12.6	41.8	20.2
10	12.9	14.8	14.3			14
10.5	14.3	17.4	17.1	25	16.2	18
11	138.7	198.6	237.7			191.7
11.5	66.3	42.7	82.7	72.4		66.1
12.5	32.1	29				30.6
13.5	44	51.9				48
14.5	67.7	91.3	27.6			62.2
15.5	18.5	34.8	16.8			23.4
16.5	25	19.8				22.4
17.5	21.3	27				29

bioRxiv preprint doi: https://doi.org/10.1101/2023.10.06.561279; this version posted October 13, 2023. The copyright holder for this preprint (which was not certified by peer review) is the author/funder, who has granted bioRxiv a license to display the preprint in perpetuity. It is made available under aCC-BY 4.0 International license.

Figure 3 __ Source data 3. Primers used for RT-PCR.

**gene**	**sequence accession number (NCBI)**	**forward primer (5'-3')**	**reverse primer (5'-3')**
Met	JN416986.1	TACTCCATCCACACAGTCAAGG	TTCCGTGATTCGACGATCTCTC
Kr-h1	JN416989.1	ACTCCGTCGAATGGTACTAGTG	GTTCTTGCATGGGAGGAAACTG
myo	GASN02042720.1	TTCAACAAGCAAGCCCACAAG	ACACCGATCCACTACCATTCC
rp49	AB689035.1	CTAAAGAGGAACTGGCGCAAAC	GTTTAGTCTTCTTGGCGCTTCC

bioRxiv preprint doi: https://doi.org/10.1101/2023.10.06.561279; this version posted October 13, 2023. The copyright holder for this preprint (which was not certified by peer review) is the author/funder, who has granted bioRxiv a license to display the preprint in perpetuity. It is made available under aCC-BY 4.0 International license.

Figure 4 __ Source Data 1. The progression of embryonic development of embryos treated with solvent or 7-ethoxyprecocene (7EP) at 3.5 days of development. Developmental rescue was attempted by subsequent treatment with a juvenile hormone mimic (pyriproxyfen) at the indicated developmental time thereafter. air: air appears between embryo and eggshell because of resorption of extraembryonic fluid; eye: appearance of eye pigment. Percent values are based on surviving embryos. *: these embryos blocked before dorsal closure because of the early timing of treatment with pyriproxyfen.

treatment	#	d 5.5	d 6.5	d 7.5	d 8.5	d 9.5	d 10.5	d 11.5	d 12.5	d 13.5
none	30	eye: 0	eye: 23 [77%]	eye: 29 [97%]	air: 0 [0%]; dead: 1	air: 8 [28%]	air: 27 [93%]	air: 29 [100%]	hatch: 21 [72%]	
cyclohexane (d3.5)	30	eye: 0	eye: 26 [87%]	eye: 30 [100%]	air: 0 [0%]	air: 23 [77%]; dead: 2	air: 28 [100%]	air: 28 [100%]; hatch: 4 [14%]	hatch: 28 [100%]	
1 ug 7EP (d3.5)	30	eye: 0	eye: 30 [100%]	eye: 30 [100%]	air: 0 [0%]; dead: 1	air: 2 [7%]	air: 3 [10%]; dead: 1	air: 4 [14%]; hatch: 1 [3%]	air: 4 [14%]; hatch: 3 [11%]	
1 ug 7EP [d3.5]; 1ng Pyri [d6.5]	15	eye: 0	eye: 13 [87%]	eye: 30 [100%]	air: 0 [0%]*	air: 0 [0%]*	air: 0 [0%]*	air: 0 [0%]*	air: 0 [0%]*	
1 ug 7EP [d3.5]; 1ng Pyri [d7.5]	30	eye: 0	eye: 27 [90%]	eye: 29 [97%]	air: 1 [3%]; dead: 1	air: 11 [38%]	air: 17 [57%]	air: 17 [57%]; hatch: 10 [34%]	air: 17 [57%]; hatch 12 [41%]	
1 ug 7EP [d3.5]; 1ng Pyri [d8.5]	30	eye: 0	eye: 22 [73%]; dead: 1	eye: 27 [93%]	air: 0 [0%]	air: 16 [55%]	air: 29 [100%]	air: 29 [100%]; hatch: 1 [3%]	air: 29 [100%]; hatch: 27 [93%]	
1 ug 7EP [d3.5]; 1ng Pyri [d9.5]	30	eye: 0	eye: 27 [90%]	eye: 27 [90%]; dead: 1	air: 0 [0%]	air: 0 [0%]	air: 14 [50%]	air: 26 [93%]; hatch: 0 [0%]	air: 26 [93%]; hatch: 5 [18%]	hatch: 26 [93%]

bioRxiv preprint doi: https://doi.org/10.1101/2023.10.06.561279; this version posted October 13, 2023. The copyright holder for this preprint (which was not certified by peer review) is the author/funder, who has granted bioRxiv a license to display the preprint in perpetuity. It is made available under aCC-BY 4.0 International license.

Figure 6 ___ Source data 1. The relationship of the time of treatment with a JH mimic (1 ng pyriproxyfen) to when embryonic development subsequently stalled. EEF: extraembryonic fluid; pre-dorsal closure was evident by dorsal closure to the neck region, but the posterior head capsule had not fully formed.

stage attained	N	germband	extended limb buds	post katatrepsis	eye pigment	pre-dorsal closure	dorsal closure	resorb EEF	hatch
age at treatment with JHm									
0.5d	16	12 [75%]	9 [56%]						
1.5d	18	18 [100%]	18 [100%]						
2.5d	20	20 [100%]	20 [100%]						
3.5d	21	21 [100%]	21 [100%]	21 [100%]	11 [52%]				
4.5d	21	21 [100%]	21 [100%]	21 [100%]	21 [100%]	21 [100%]	0 [0%]		
5.5d	15	15 [100%]	15 [100%]	15 [100%]	15 [100%]	14 [94%]	1 [6%]		
6.5d	19	19 [100%]	19 [100%]	19 [100%]	19 [100%]	19 [100%]	19 [100%]	15 [79%]	13 [68%]
7.5d	15	15 [100%]	15 [100%]	15 [100%]	15 [100%]	15 [100%]	15 [100%]	15 [100%]	15 [100%]

bioRxiv preprint doi: https://doi.org/10.1101/2023.10.06.561279; this version posted October 13, 2023. The copyright holder for this preprint (which was not certified by peer review) is the author/funder, who has granted bioRxiv a license to display the preprint in perpetuity. It is made available under aCC-BY 4.0 International license.

Figure 7 ___ Source data 1. The effects of early treatment with a JH mimic (1 ng pyriproxyfen) on the subsequent proliferative activity in the embryonic limb buds indicated by the number of limb cells expressing phosphor histone H3 (pPH3). N is the number of embryos scored for each treatment.

Treatment	score embryos	N	Average number of pHH3 positive cells/limb	average
**Controls: treat at 1.5 d AEL**				
cyclohexane 1.5 d	2.5 d AEL	3	11.3, 12.7, 9	11
cyclohexane 1.5 d	3.5 d AEL	13	12.8, 17.6, 13.5, 17.7, 16. 10, 15, 18, 12.5, 22, 12, 16, 16.5.	15.3
cyclohexane 1.5 d	4.5 d AEL	11	21.3, 29.2, 22.5, 24, 25, 29.3, 23, 24.3, 23, 26, 33.5	25.6
cyclohexane 1.5 d	5.5 d AEL	10	20, 22.3, 21, 20.3, 22.7, 27.3, 25.7, 31, 29, 19.5	23.9
cyclohexane 1.5 d	6.5 d AEL	6	11.3, 11, 14, 10.6, 15.3, 14.3	12.8
cyclohexane 1.5 d	7.5 d AEL	4	7.3, 8, 7.3, 7, 3	7.5
**JHm: treat at 1.5 d AEL**				
JHm 1.5 d	2.5 d AEL	3	14, 13, 10.3	12.4
JHm 1.5 d	3.5 d AEL	11	17, 15.5, 16.4, 14.7, 16.7, 15.5, 4.8, 13.3, 14, 11, 5.8	13.2
JHm 1.5 d	4.5 d AEL	11	2.7, 3.7, 3.2, 1., 0., 1.7, 0.3, 0, 1.5, 1.7, 0.7	1.5
JHm 1.5 d	5.5 d AEL	13	7, 9, 4.5, 6.3, 2, 5.3, 2, 0.3, 0, 0.3, 0.2, 3.3, 0	3.1
JHm 1.5 d	6.5 d AEL	14	5.3, 0, 4.3, 0.6, 1.5, 0.6, 1.5, 0, 0, 0, 0.3, 0, 4, 1.6, 0	1.4
JHm 1.5 d	7.5 d AEL	8	0,0.7, 0, 0.7, 0, 0.3, 0, 0,	0.2
